# The Exported Protein PbCP1 Localises to Cleft-Like Structures in the Rodent Malaria Parasite *Plasmodium berghei*

**DOI:** 10.1371/journal.pone.0061482

**Published:** 2013-04-26

**Authors:** Silvia Haase, Eric Hanssen, Kathryn Matthews, Ming Kalanon, Tania F. de Koning-Ward

**Affiliations:** 1 School of Medicine, Deakin University, Waurn Ponds, Victoria, Australia; 2 Electron Microscopy Unit, Bio21 Institute, University of Melbourne, Parkville, Victoria, Australia; State University of Campinas, Brazil

## Abstract

Protein export into the host red blood cell is one of the key processes in the pathobiology of the malaria parasite *Plasmodiumtrl falciparum*, which extensively remodels the red blood cell to ensure its virulence and survival. In this study, we aimed to shed further light on the protein export mechanisms in the rodent malaria parasite *P. berghei* and provide further proof of the conserved nature of host cell remodeling in *Plasmodium spp*. Based on the presence of an export motif (R/KxLxE/Q/D) termed PEXEL (*Plasmodium* export element), we have generated transgenic *P. berghei* parasite lines expressing GFP chimera of putatively exported proteins and analysed one of the newly identified exported proteins in detail. This essential protein, termed PbCP1 (*P. berghei* Cleft-like Protein 1), harbours an atypical PEXEL motif (RxLxY) and is further characterised by two predicted transmembrane domains (2TMD) in the C-terminal end of the protein. We have functionally validated the unusual PEXEL motif in PbCP1 and analysed the role of the 2TMD region, which is required to recruit PbCP1 to discrete membranous structures in the red blood cell cytosol that have a convoluted, vesico-tubular morphology by electron microscopy. Importantly, this study reveals that rodent malaria species also induce modifications to their host red blood cell.

## Introduction

The human malaria parasite *Plasmodium falciparum* exports approximately three hundred proteins into its host red blood cell (RBC) [1] leading to a number of morphological alterations to the cell such as increased rigidity and adhesiveness (for review, see [2]). Small electron-dense knob-like structures protrude from the RBC surface [3] and act as platforms from which the major virulence determinant and cytoadherence factor PfEMP1 (*P. falciparum* erythrocyte membrane protein 1) is presented [4], [5]. Furthermore, parasite-induced membranous structures appear in the host cell cytoplasm, including the Maurer's clefts, which have been implicated to be involved in sorting and trafficking of virulence proteins (for reviews, see [6], [7]) and the HSP40-containing J-dots [8]. These alterations to the RBC not only enable the pathogen to resist the febrile episodes of the host and to acquire nutrients from the host serum, they also facilitate adherence of the infected RBC (iRBC) to the vascular endothelium, allowing the parasite to avoid immune clearance, and hence securing the survival of the parasite.

Since the mature RBC is devoid of any secretory system, the parasite has had to establish its own trafficking machinery to export its proteins from the endoplasmatic reticulum (ER), beyond its own boundaries across the parasitophorous vacuole (PV) and parasitophorous vacuole membrane (PVM) into the host RBC. Passage of most exported proteins across the parasite-host cell interface is mediated by a pentameric amino acid motif (R/KxLxE/Q/D), termed PEXEL (*Plasmodium* export element) or HT (host targeting) motif, which is located downstream of an N-terminal signal peptide [9], [10]. Cleavage of this motif selectively enables these proteins to cross the parasite-host cell boundary through a putative *Plasmodium* translocon of exported proteins (PTEX) [11], [12]. Whilst both the PEXEL motif and PTEX are conserved amongst *Plasmodium spp* [1], [12], little is known about protein export and host cell modifications in other malaria parasite species as previous studies have focused predominantly on *P. falciparum*. However, to investigate the functional significance of protein export in malaria pathogenesis, it is essential to study this in the context of the host by using *Plasmodium* species for which animal models exist. Recent studies have provided the first evidence of PEXEL-mediated export of *P. falciparum* proteins in the rodent malaria parasite *P. berghei* and of export of rodent parasite proteins in a PEXEL-dependent and -independent manner [13]–[15]. Moreover, the finding that *P. berghei* ANKA parasites indeed sequester [16] suggest common host cell remodeling strategies may be utilised amongst *Plasmodium spp* to ensure parasite virulence and survival.

Thus, in order to gain a deeper insight and understanding into the evolution of protein export and its importance in malaria pathogenesis, we aimed to further evaluate the rodent ‘exportome’ [1] by identifying novel exported proteins in the rodent malaria parasite *P. berghei* and the mechanisms by which they are exported. From this, we focused on one protein, termed PbCP1 (***P*** *.* ***berghei*** **C**left-like **P**rotein **1**), which localises to discrete punctuate structures in the RBC cytosol. We have verified the role of the PEXEL motif in PbCP1 export and show that the putative two transmembrane domain region (2TMD) of PbCP1 is required to promote trafficking to the newly identified structures in the RBC cytosol. Electron microscopy (EM) reveals discrete parasite-induced structures of a membranous nature with immuno-EM confirming localisation of PbCP1 to these structures. Unsuccessful attempts to generate a *pbcp1* knockout parasite line suggest this protein plays an important role in the pathobiology of *P. berghei*. In summary, our findings indicate a conserved nature of host cell remodeling within *Plasmodium spp*, which may help to refine the export processes in the rodent malaria parasite *P. berghei*. This insight is necessary in establishing *P. berghei* as a protein export model, which would ultimately allow the expression and trafficking of *P. falciparum* virulence proteins in chimeric *P. berghei*-iRBC to investigate their contribution to parasite virulence in an isolated setting.

## Results

### PEXEL export in *P. berghei*

In order to verify the rodent ‘exportome’ [1] and identify exported proteins in the rodent malaria parasite *P. berghei*, predicted *P. berghei* PEXEL proteins with a minimum ExportPred score of ‘4’ [1] were derived from the PlasmoDB database (PlasmoDB 6.0 and Table S1). From this, 37 putatively exported proteins containing six PEXEL variants were identified, those being RxLxE (17 proteins), RxLxD (9 proteins), RxLxY (7 proteins), RxLxS (2 proteins), KxLxS (1 protein) and KxLxE (1 protein) (Table S1). Ten were selected for further analysis in this study; seven proteins were chosen from the ‘typical’ RxLxE and RxLxD PEXEL motif groups, as these motifs have been demonstrated to mediate export in *P. falciparum* [9], [10]. A further three proteins were selected from the next most abundant PEXEL group containing an RxLxY motif, which appears to be unique to the rodent species and, to our knowledge, has not been validated to mediate export in any *Plasmodium spp*. All ten candidates are encoded by two exons, a common feature shared amongst many *P. falciparum* exported proteins [1], and are characterised by the presence of a signal peptide or a hydrophobic stretch in the N-terminus upstream of the PEXEL motif [9], [10] (Table S1). With the exception of one soluble protein containing an RxLxE motif, the remaining candidates harbour either one or two putative TMDs, a feature present in the majority of the 37 proteins identified. Finally, amongst the RxLxE, RxLxD and RxLxY groups, orthologues of members of the *P. yoelli* subtelomeric family PYST-B proteins could be identified, five of which were included in this study (Table S1).

**Figure 1:**
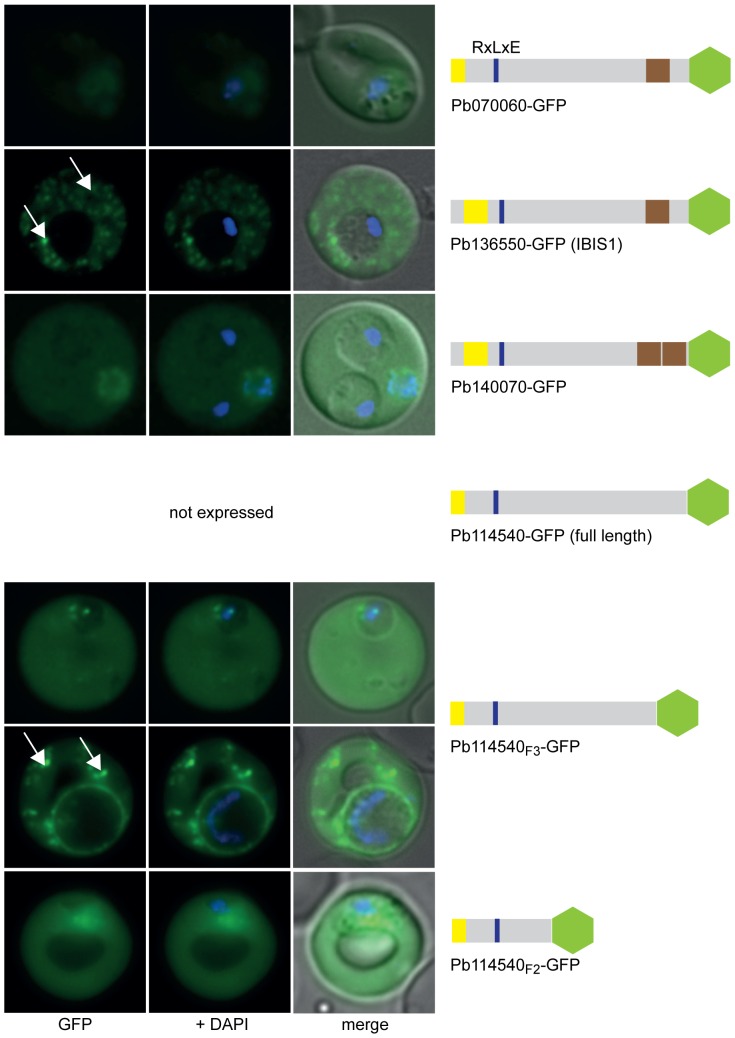
RxLxE GFP chimeras are exported into the RBC cytosol. Pb070060, Pb136550 and Pb140070 were exported into the RBC cytosol. Full-length expression of Pb114540-GFP could not be obtained, however, two shorter versions (Pb114540_F3_ and Pb114540_F2_) were expressed and exported into the host cell cytosol. Pb136550-GFP and Pb114540_F3_-GFP also localised to discrete punctuate structures (indicated by white arrows) in the RBC cytosol. GFP fluorescence is indicated by GFP (green), parasite nuclei are stained with DAPI (blue) and merged images include the bright field. A schematic structure of all GFP chimera is depicted beside the panel: signal peptide/hydrophobic stretch (yellow), PEXEL (blue), predicted TMD region (brown) and GFP (green).

**Figure 2:**
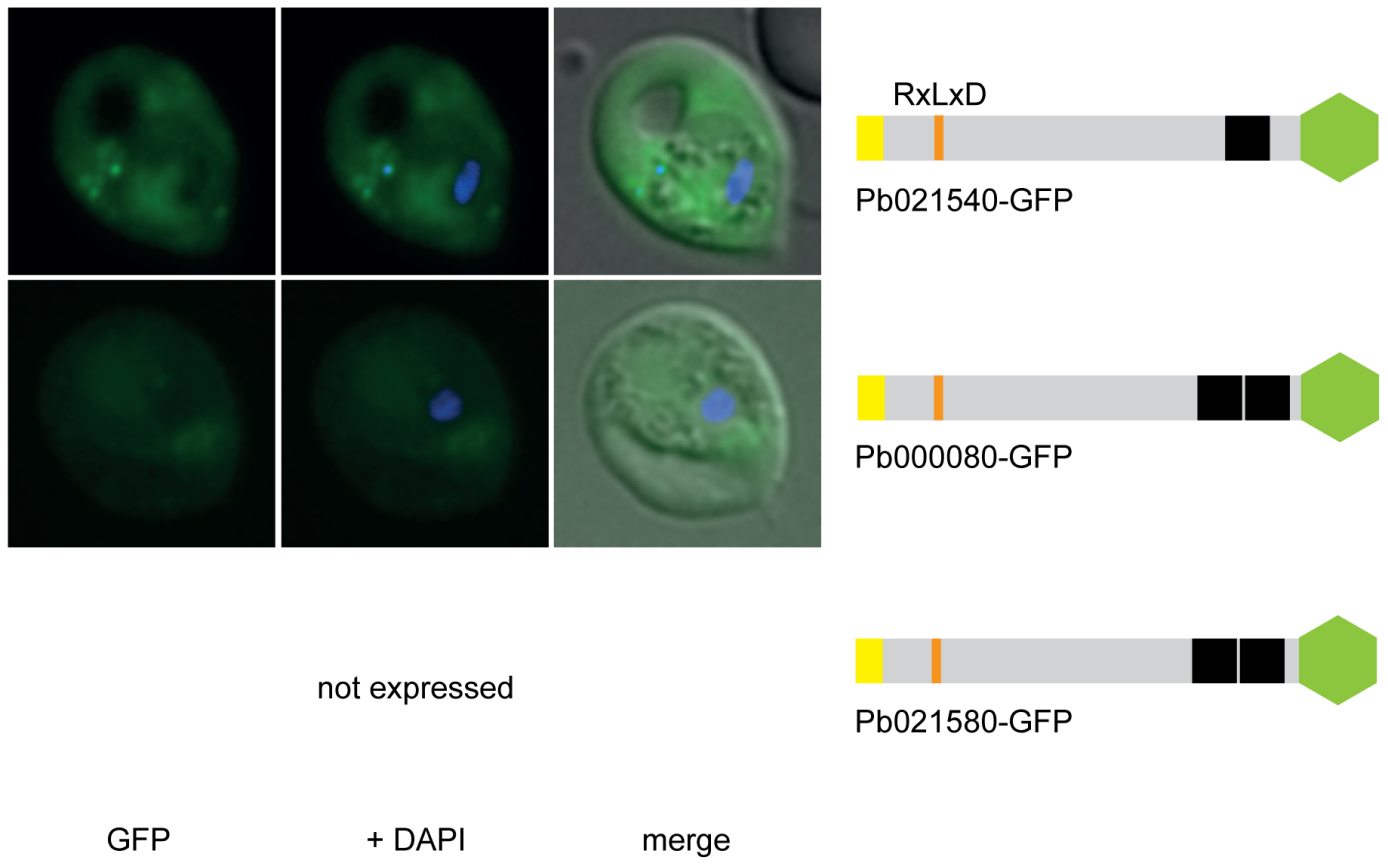
RxLxD containing proteins Pb021540-GFP and Pb000080-GFP are exported into the RBC cytosol. Pb021580-GFP was not expressed in two independent transgenic parasite lines. GFP fluorescence is indicated by GFP (green), parasite nuclei are stained with DAPI (blue) and merged images include the bright field. A schematic structure of all GFP chimera is depicted beside the panel: signal peptide/hydrophobic stretch (yellow), PEXEL (orange), predicted TMD region (black) and GFP (green).

**Figure 3:**
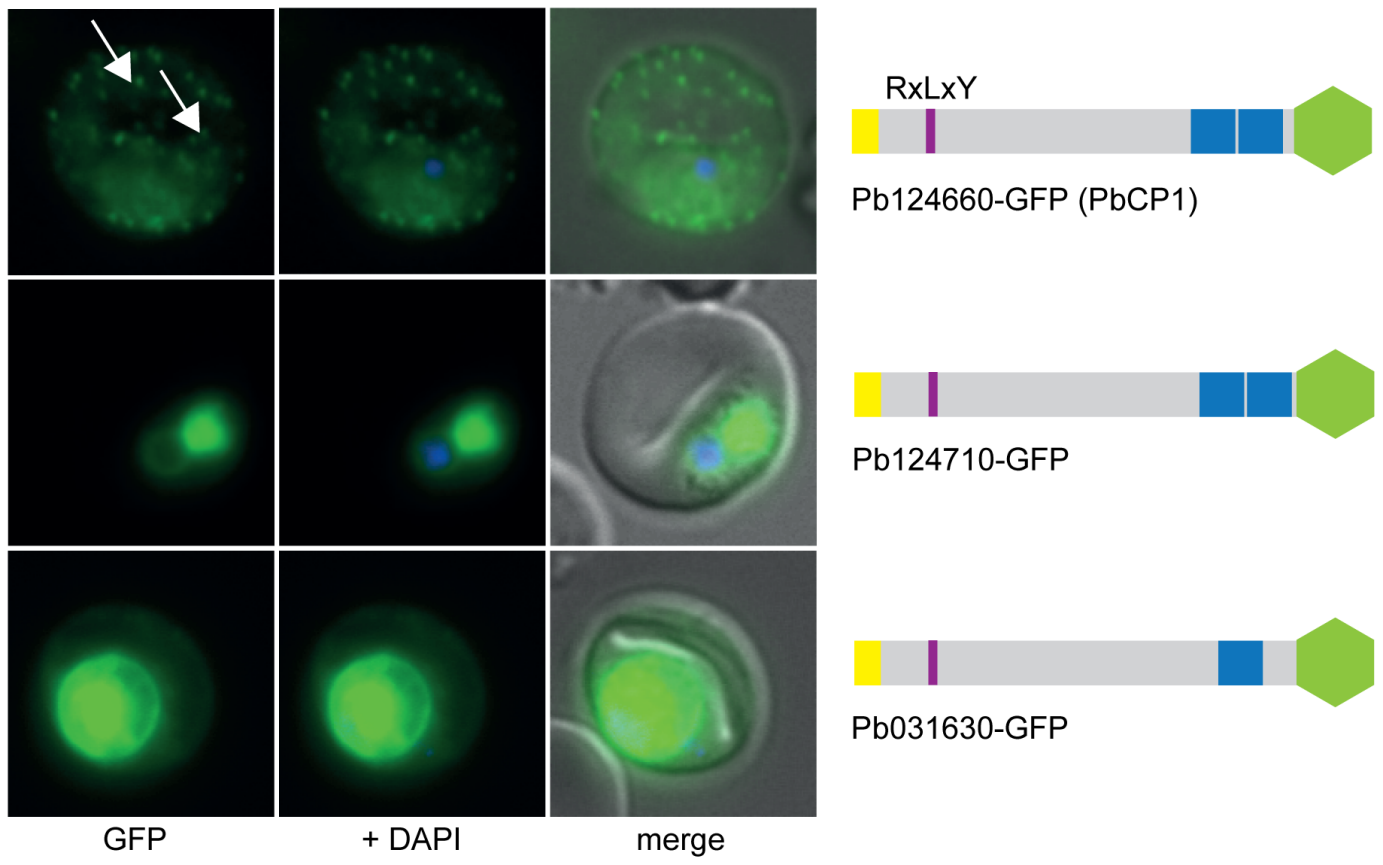
All RxLxY containing proteins are exported into the RBC. Lower expression levels in the RBC cytosol and accumulation of GFP chimera at the ER and/or PV(M) could also be observed for Pb124710-GFP and Pb031630-GFP. Pb124660-GFP (PbCP1) was also found to localise to extra-parasitic structures within the RBC cytosol (white arrows). GFP fluorescence is indicated by GFP (green), parasite nuclei are stained with DAPI (blue) and merged images include the bright field. A schematic structure of all GFP chimera is depicted beside the panel: signal peptide/hydrophobic stretch (yellow), PEXEL (purple), predicted TMD region (blue) and GFP (green).

Next, transgenic *P. berghei* ANKA (PbANKA) parasite lines expressing GFP chimera of the selected putatively exported proteins were generated and imaged by live fluorescence microscopy to examine the localisation of the expressed reporter proteins (Figures 1, 2, 3 and Table S2). This analysis revealed that three of the four RxLxE containing proteins (Pb070060-GFP, Pb136550-GFP and PB140070-GFP) could indeed be exported (Figure 1). As Pb070060-GFP displayed only faint fluorescence during live microscopy, export was subsequently confirmed by immunofluorescence analysis (IFA) of acetone fixed iRBCs using anti-GFP antibodies (Figure S1A). However, live microscopy, IFA and Western blot analysis could not confirm any expression of the fourth protein in this group (full length Pb114540-GFP), which represented the only soluble candidate, in two independent transgenic parasite lines. Therefore, two shorter versions of Pb114540-GFP were generated (with Pb114540_F3_ including aa_1–411_ and Pb114540_F2_ encompassing aa_1–183_, respectively) and both GFP chimera were exported into the RBC cytosol (Figure 1). Out of the second group, comprising putatively exported proteins with RxLxD as a PEXEL motif, the putative TMD-containing proteins Pb021540-GFP and Pb000080-GFP were found to localise to the RBC cytosol (Figure 2). Similarly to Pb114540-GFP, expression of Pb021580-GFP could not be detected in two independent transfections of this construct. Lastly, all RxLxY containing proteins were exported into the RBC, however, lower expression levels in the RBC cytosol and accumulation of GFP chimera at the ER and/or PV(M) could also be observed for Pb124710-GFP and Pb031630-GFP (Figure 3). IFA of acetone fixed iRBCs confirmed the localisation of Pb124710-GFP in the RBC cytosol (Figure S1A). Lastly, a recent study by Pasini et al. confirmed the localisation of PbANKA_021540 and PbANKA_114540 and attempts to genetically disrupt PbANKA_070060 demonstrated this protein to be non-essential [17].

Interestingly, live cell imaging revealed that three candidates, those being Pb124660-GFP, hereafter referred to as ***P*** *.* ***berghei*** **C**left-like **P**rotein **1** (PbCP1), Pb136550-GFP and ∼50% of parasites expressing Pb114540_F3_-GFP were not only exported into the RBC but were also found to localise to discrete punctuate structures in the RBC cytosol (indicated by white arrows, Figures 1 and 3). These punctuate structures appeared to be highly dynamic with the mobility more prominent in the early stages of parasite development (a movie of the PbCP1-GFP dynamics is shown in Movie S1). In agreement with our observations, Pb136550 (also known as IBIS1) has previously been shown to associate with similarly dynamic structures in *P. berghei* iRBCs [18]. To confirm the localisation of PbCP1, a second transgenic parasite line was generated in which the GFP reporter was replaced with a triple-haemagglutinin/streptavidin tag (3xHA/Strep). IFA of acetone/methanol fixed iRBCs displayed the same export phenotype for PbCP1-3xHA/Strep as observed for the PbCP1-GFP expressing parasite line and immunoblot analysis using anti-HA antibodies revealed a doublet protein band at the predicted MW of ∼30 kDa (indicated by asterisks, Figure S1B).

Expression of all exported GFP chimera was verified by Western blot analysis and revealed protein bands of the predicted molecular weight (data not shown), with the exception of PbCP1-GFP, for which a doublet protein band was observed around the expected size of ∼52 kDa (indicated by asterisks, Figure S2).

### The RxLxY motif in *P. berghei* is functional

**Figure 4:**
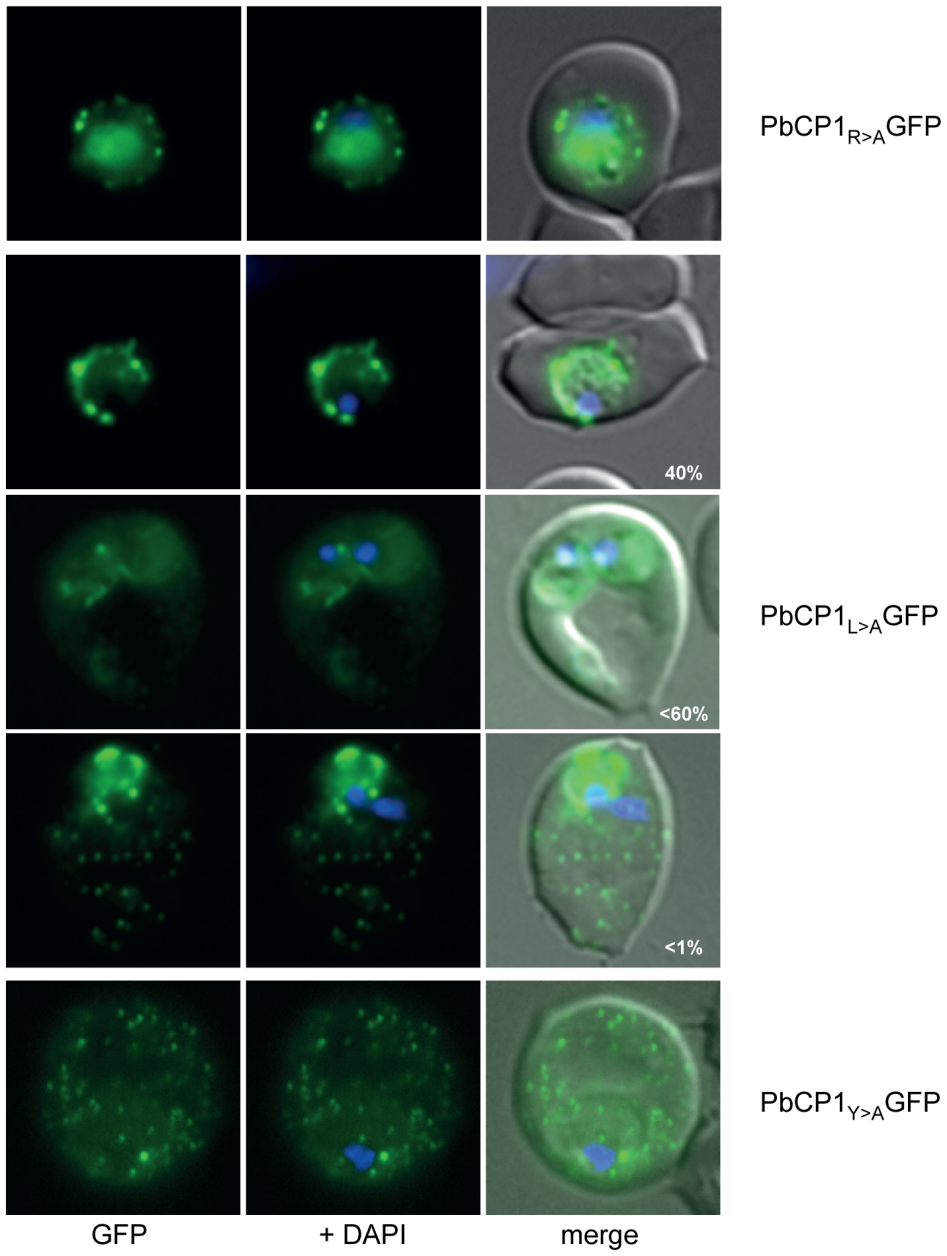
Investigation of the RxLxY PEXEL motif in PbCP1. Substitution of the first PEXEL residue (R) abolished export of PbCP1_R>A_GFP and led to the accumulation of the respective GFP chimera within the parasite and the PV(M). Different phenotypes could be observed for the PbCP1_L>A_GFP mutant. Mutation of the last PEXEL residue (Y) did not influence export of the resulting PbCP1_Y>A_GFP fusion protein to the RBC cytosol and the extra-parasitic structures.

Whilst PbCP1, Pb124710 and Pb031630 are exported, these proteins harbour an atypical PEXEL motif (RxLxY) that has not previously been validated to mediate export. Accordingly, single amino acid substitutions of the individual PEXEL residues R, L and Y (to alanine) of PbCP1 were created by site directed mutagenesis and the resulting GFP chimera were episomally expressed in PbANKA parasites. Mutation of the last PEXEL residue (Y) did not influence export of the resulting PbCP1_Y>A_GFP fusion protein to the RBC cytosol, resembling the localisation of the wild-type PEXEL-GFP chimera (Figure 4). This suggests a rather dispensable role of the tyrosine residue in the PEXEL motif of this protein. In contrast, substitution of the arginine residue abolished export of the respective PbCP1_R>A_GFP chimera and led to the accumulation of this protein within the parasite and at the parasite periphery. Whether this is at the parasite plasma membrane (PPM) (a location which has recently been shown to play a crucial role in the translocation of TMD containing proteins [19] or the PV(M) could not be definitely determined by live cell imaging. Only a small proportion (<1%) of parasites were capable of exporting the R>A mutant into the RBC cytosol. Different phenotypes could also be observed for the L>A mutant PbCP1-GFP chimera; in ∼40% of the expressing parasites, PbCP1_L>A_GFP was not exported and accumulated at the PPM and/or PV(M), less than 60% exported this GFP fusion protein into the RBC cytosol, and only a small percentage displayed PbCP1-GFP wild-type localisation (Figure 4). Taken together, these results indicate that the conserved arginine PEXEL residue and, albeit to a lower extent the leucine residue, are necessary for efficient export, a finding that has also been described in *P. falciparum* [20], [21]. It should be noted that similar to the wild-type protein, doublet protein bands for all PbCP1 PEXEL mutants could be detected by Western blot analysis (Figure S2).

### Trafficking of PbCP1 to the discrete structures in the RBC cytosol requires the 2TMD region

**Figure 5:**
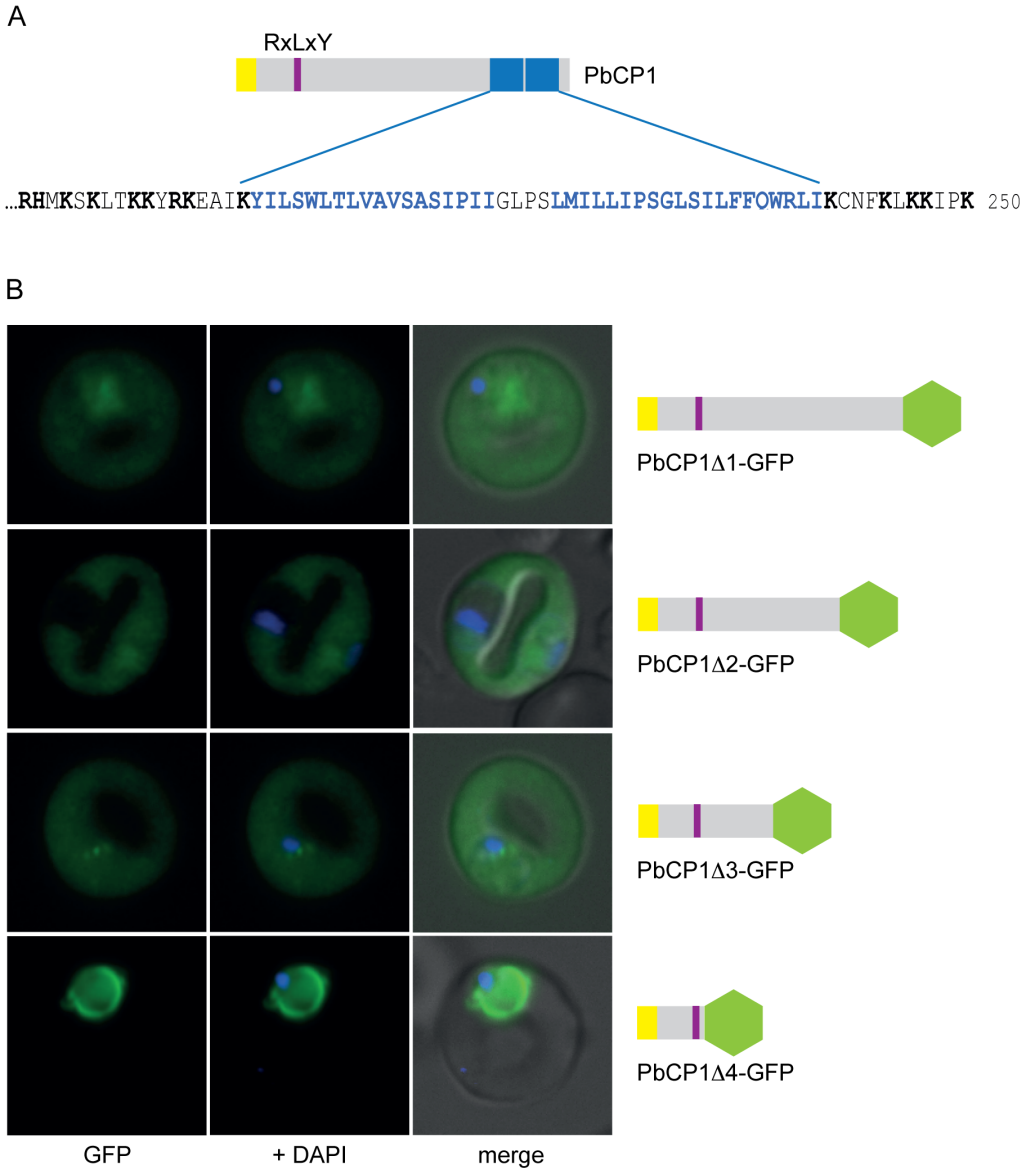
Dissecting the trafficking requirements of PbCP1. (A) Extract of the PbCP1 C-terminus depicting the predicted 2TMD region (blue and bold), which is flanked by several positively charged residues (black and bold). (B) Consecutive 50aa truncations of PbCP1 were undertaken and the localisation of the respective GFP chimera analysed in live PbANKA parasites. Deletion of the 2TMD region abolished export to the punctuate structures in the respective PbCP1Δ1-GFP chimera, but export into the RBC cytosol was unaffected. Further truncations of 100aa (PbCP1Δ2-GFP) and 150aa (PbCP1Δ3-GFP), respectively, had no effect on export of the corresponding GFP fusion proteins. In the 200aa deletion, GFP is fused directly to the PEXEL motif and led to the accumulation of PbCP1Δ4-GFP within the parasite and the parasite periphery.

The C-terminal end of PbCP1 is characterised by two predicted TMDs (aa_198–215_ and aa_220–239_), which are separated by a short loop of only 4 residues (Figure 5A). The 2TMD architecture is a commonly shared protein feature within the 2TMD superfamily of *P. falciparum*, including the RIFINs, STEVORs and PfMC-2TM proteins [22]–[24], indicating a conserved protein domain structure amongst *Plasmodium* spp. The observed export phenotype of PbCP1-GFP encouraged us to further analyse the trafficking requirements of PbCP1 to the extra-parasitic punctuate structures. Therefore, 50aa truncations of PbCP1 were performed and the localisation of the respective GFP chimera analysed in live PbANKA parasites.

As hypothesised, deletion of the putative 2TMD region ablated trafficking of the respective PbCP1Δ1-GFP chimera to the extra-parasitic structures, however, export into the RBC cytosol was unaffected (Figure 5B). Further truncations of 100aa (PbCP1Δ2-GFP) and 150aa (PbCP1Δ3-GFP), respectively, had no effect on the export phenotype, suggesting that these regions do not contain specific information to traffic PbCP1 into the RBC cytosol (Figure 5B). However, a 200aa truncation of PbCP1, in which GFP is fused directly to the PEXEL motif, resulted in the accumulation of PbCP1Δ4-GFP within the parasite and parasite periphery (Figure 5B). This is consistent with observations made in *P. falciparum* [10], whereby the region directly flanking the PEXEL motif is needed to ensure appropriate PEXEL recognition and cleavage [20], [21]. In addition, deletion of either putative TMD showed a similar phenotype to PbCP1Δ1-GFP, with the resulting GFP chimera PbCP1ΔTMD1-GFP and PbCP1ΔTMD2-GFP being exported into the RBC cytosol but not localising to the discrete structures (Figure S3A). This further indicates that elements within both predicted TMDs are required to traffic PbCP1 to these structures. A minority (∼5%) of the parasites did not export PbCP1ΔTMD1-GFP into the RBC cytosol (Figure S3A). Expression of all truncated PbCP1-GFP chimera was also confirmed by immunoblotting using anti-GFP antibodies, which recognised protein bands of the predicted size for all proteins except for PbCP1ΔTMD1-GFP, running at an aberrant molecular weight (Figure S3B).

### The PbCP1 2TMD region contains specific trafficking information

**Figure 6:**
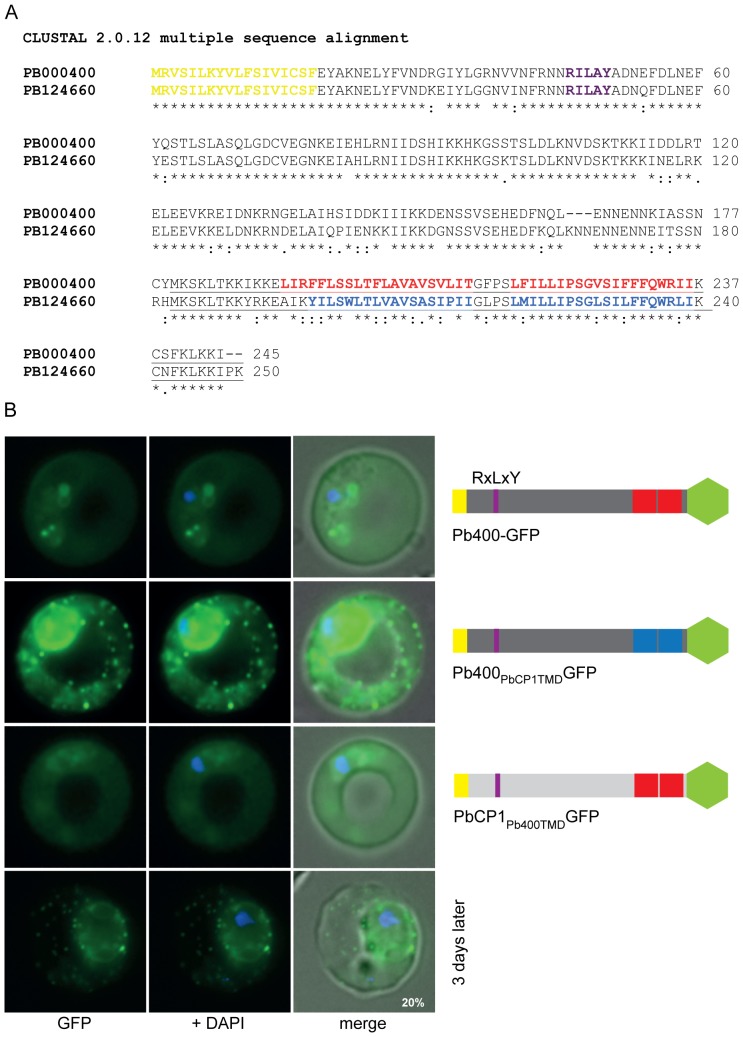
The PbCP1 2TMD region mediates trafficking to the discrete punctuate structures in the RBC cytosol. (A) Alignment of PbCP1 and its paralogue Pb400 shows 77% identity. The signal peptide is highlighted in yellow, the PEXEL motif in purple and the predicted 2TMD region in red (Pb400) and blue (PbCP1), respectively. (B) Wild-type Pb400 is episomally expressed as a GFP fusion protein and localises to the RBC cytosol in live PbANKA parasites. Replacement of the predicted Pb400 2TMD and downstream region with that of PbCP1 led to the localisation of the resulting Pb400_PbCP1TMD_GFP chimera to the discrete extra-parasitic structures in the RBC cytosol. In contrast, replacement of the putative PbCP1 2TMD and downstream region with that of Pb400 abolished trafficking of PbCP1_Pb400TMD_GFP to the punctuate structures.

The finding that both predicted TMDs are required to recruit PbCP1 to the discrete regions in the RBC cytosol led us to search for other homologous proteins that might have a similar localisation in *P. berghei*. To do so, a Blast analysis (PlasmoDB 6.0) of PbCP1 was performed and revealed 27 paralogous proteins in *P. berghei*. Orthologues could also be found in the other rodent malaria parasites, *P. yoelii* and *P. chabaudi*, but not in any of the primate *Plasmodium* species. The paralogue with the highest degree of homology (with 77% identity and 80% positives) to PbCP1 is PbANKA_000400 (Figure 6A), hereafter referred to as Pb400. However, transgenic parasites expressing full length Pb400-GFP revealed weak GFP fluorescence and export of the fusion protein into the RBC cytosol but no localisation to the discrete punctuate structures as observed in the PbCP1-GFP expressing parasite line (Figure 6B). Whilst the sequence downstream of the predicted 2TMD region of Pb400 is highly conserved to PbCP1, it lacks the terminal proline and lysine residue (Figure 6A) but inclusion of these two residues in addition to a triple-glycine linker between the C-terminus and the GFP did not influence the export phenotype nor the expression level of the resulting Pb400_PK3xG_-GFP chimera (Figure S4A). However, replacement of the GFP reporter with a smaller triple-haemagglutinin/streptavidin tag led to a similar expression pattern of the respective Pb400-3xHA/Strep fusion protein as observed for PbCP1, suggesting that the GFP reporter was masking the TMDs (Figure S4B).

Nevertheless, the phenotype of the Pb400-GFP chimera was most useful; subsequent replacement of the predicted Pb400 2TMD and downstream sequence with that of PbCP1 altered the localisation of the fusion protein from being soluble in the RBC cytosol to now localising to the discrete punctuate structures in the RBC cytosol (Figure 6B). Interestingly, this substitution greatly improved the expression levels of the Pb400_PbCP1TMD_GFP chimera relative to Pb400-GFP, being similar to that of PbCP1-GFP. Prominent GFP fluorescence at the ER and the parasite periphery could also be observed in ∼50% of the parasites. This might be due to topological changes of Pb400_PbCP1TMD_GFP caused by two additional positive charges in the new Pb400 C-terminus versus the Pb400 N-terminus. Positively charged residues flanking TMD regions are important topological determinants in membrane proteins and the substitution of a single positive charge has been shown to greatly affect the orientation of integral membrane proteins [25], [26]. In contrast, replacement of the PbCP1 2TMD and C-terminal region with the respective region of Pb400 abolished localisation of PbCP1_Pb400TMD_GFP to the punctuate structures as expected, with a soluble export phenotype in the RBC cytosol now observed (Figure 6B). Three days later, however, a proportion of parasites (∼20%) were able to traffic PbCP1_Pb400TMD_GFP to the extra-parasitic structures. Together, these experiments indicate that the putative 2TMD and C-terminal region of PbCP1 contains important information for targeting this protein to the discrete punctuate structures in the RBC cytosol. Expression of all GFP fusion proteins was verified by Western blot analysis detecting protein bands of the predicted size of ∼52 kDa, however, interestingly a doublet protein band (indicated by asterisks, Figure S5) could now be detected for Pb400_PbCP1TMD_GFP in contrast to wild-type Pb400-GFP.

### The TMD region of IBIS1 is not sufficient to mediate export to the extra-parasitic structures

**Figure 7:**
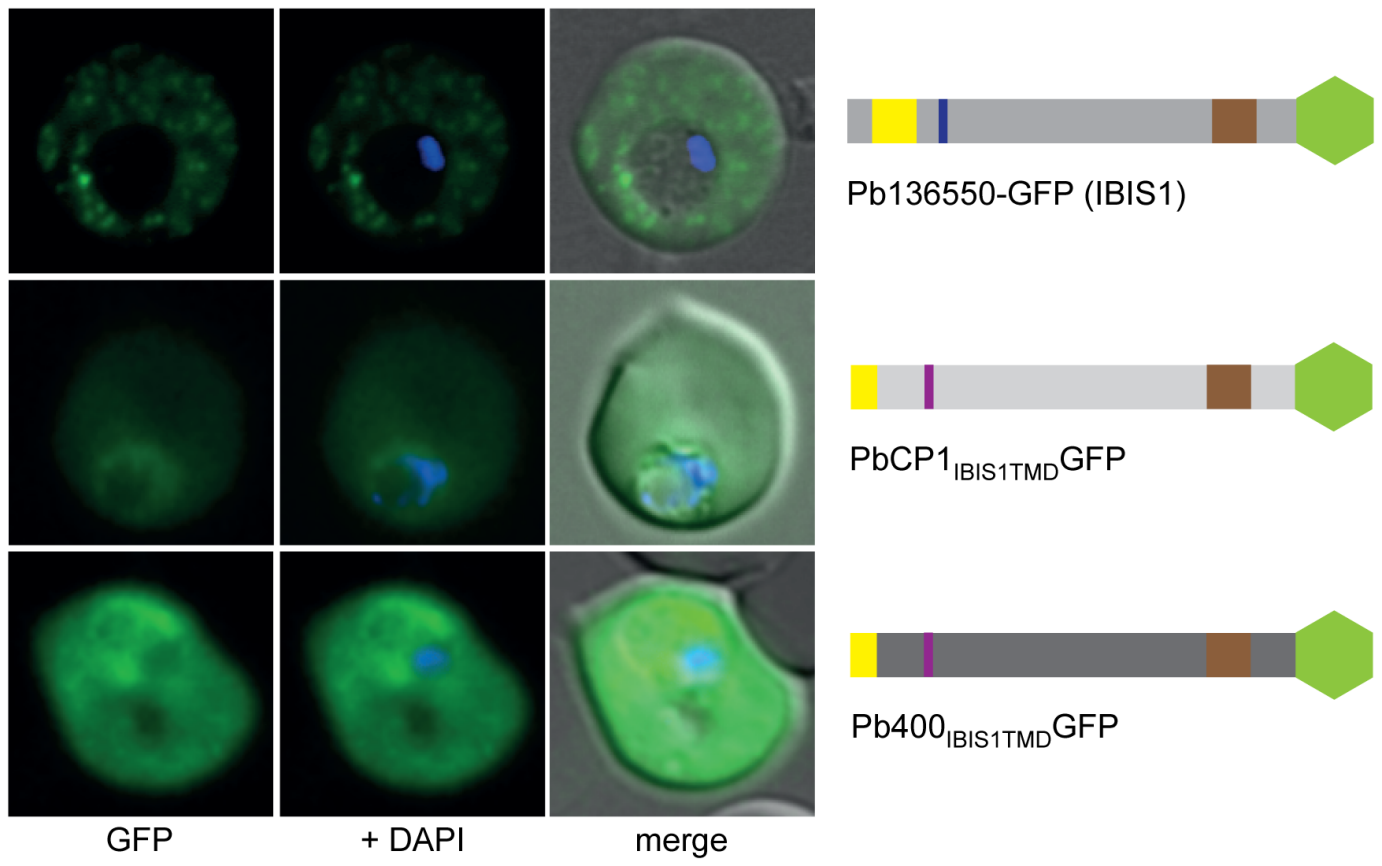
The TMD region of IBIS1 is not sufficient to mediate trafficking to the extra-parasitic structures. The predicted PbCP1 and Pb400 2TMD and downstream region, respectively, were replaced with the single TMD (highlighted in brown in the schematic structure beside the panel) and downstream region of IBIS1 (17). The resulting fusion proteins PbCP1_IBIS1TMD_GFP and Pb400_IBIS1TMD_GFP, however, did not display the punctuate localisation pattern as seen in wild-type IBIS1-GFP and were only exported into the RBC cytosol.

Our initial screen for exported proteins in *P. berghei* revealed a second protein with a similar export phenotype to PbCP1, that being the recently described Pb136550 protein known as IBIS1 [18]. However, the protein structure of IBIS1 differs not only in its PEXEL motif but also in the presence of a single TMD region in the C-terminal half of the protein. Based on our findings for PbCP1, we hypothesised a similar role of the Pb136550 TMD region in trafficking of IBIS1 to the discrete structures that Ingmundson *et al*. [18] observed in the RBC cytosol. Therefore, we replaced the putative PbCP1 2TMD and C-terminal region with the single TMD and downstream region of IBIS1. Whilst the resulting GFP chimera PbCP1_IBIS TMD_GFP could still be exported, it did not display the punctuate localisation pattern as seen in wild-type IBIS1 (Figure 7A). Likewise, replacement of the predicted Pb400-GFP 2TMD and downstream region with the single TMD and C-terminal region of IBIS1 did not change its phenotype within the RBC cytosol (Figure 7A). Together these observations suggest that additional information upstream of the IBIS1 TMD region is needed to mediate trafficking to the novel structures. Both GFP chimera were detected at the predicted MW of ∼50 kDa in immunoblots (Figure S5).

### *P. berghei* induces membranous structures in the cytosol of iRBCs

**Figure 8:**
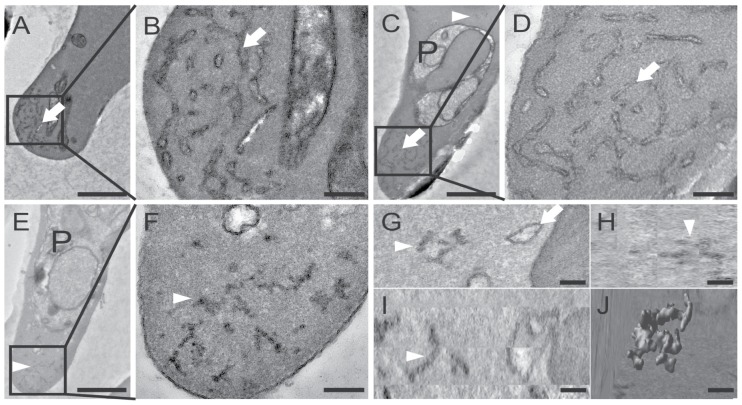
*P. berghei* induces membranous vesico-tubular structures in the RBC cytsosol. TEM reveals different membranous structures in reticulocytes: The endoplasmatic reticulum, which is characteristic for premature RBCs is indicated by white arrows in uninfected reticulocytes (A+B) and in infected reticulocytes (C+D). The parasite is indicated by ‘P’. Enlargement of the regions boxed in panels A, C and E are shown in panels B, D and F, respectively. Infected RBCs also contain condensed membranous structures within the RBC cytosol (E–I, white arrowheads). Electron tomography performed on an entire infected reticulocyte reveals the 3D architecture of the extra-parasitic membranous structure: G, H and I represent the orthoslices (xy, zy and xz, respectively) of the parasite-induced structure rendered in J. Scale bars: A, C, E 1 mm; B, D, F 200 nm and G–J 100 nm.

The observation of the discrete punctuate pattern in the cytosol of *P. berghei* iRBCs (Figure 1) indicated the presence of host cell modifications similar to the Maurer's clefts or Schueffner's dots in the human pathogens *P. falciparum* and *P. vivax*, respectively (for review, see [27]). Since at the time of the study such structures had not been described for the rodent *Plasmodium* species, *P. berghei* iRBCs were examined by transmission electron microscopy (TEM). As *P. berghei* parasites preferably invade reticulocytes, which are known to contain several remnant membranous structures, we specifically examined infected and uninfected reticulocytes as a control to exclude any mis-interpretation of our findings. Electron micrographs of uninfected (Figure 8A–B) and infected reticulocytes (Figure 8C–D) display a distinct morphology of the prominent reticular structures in premature RBCs (indicated by white arrows) compared to the small, condensed membranous structures (indicated by white arrowheads, Figure 8E–I), which could only be detected in the cytosol of iRBCs. These parasite-induced structures appear to have the same condensed morphology and are similar in size to each other (∼200 nm). In addition, electron tomograms of serial sections of *P. berghei* iRBCs provided further evidence that the discrete membranous structures are indeed independent entities and not connected to the reticular network (Movie S2). A 3D reconstruction of one of the *P. berghei*-induced structures reveals a convoluted, vesico-tubular morphology (Figure 8J) compared to the flattened cisternae as described for the Maurer's clefts in *P. falciparum* [28].

### PbCP1 is membrane associated and localises to the parasite-induced structures

**Figure 9:**
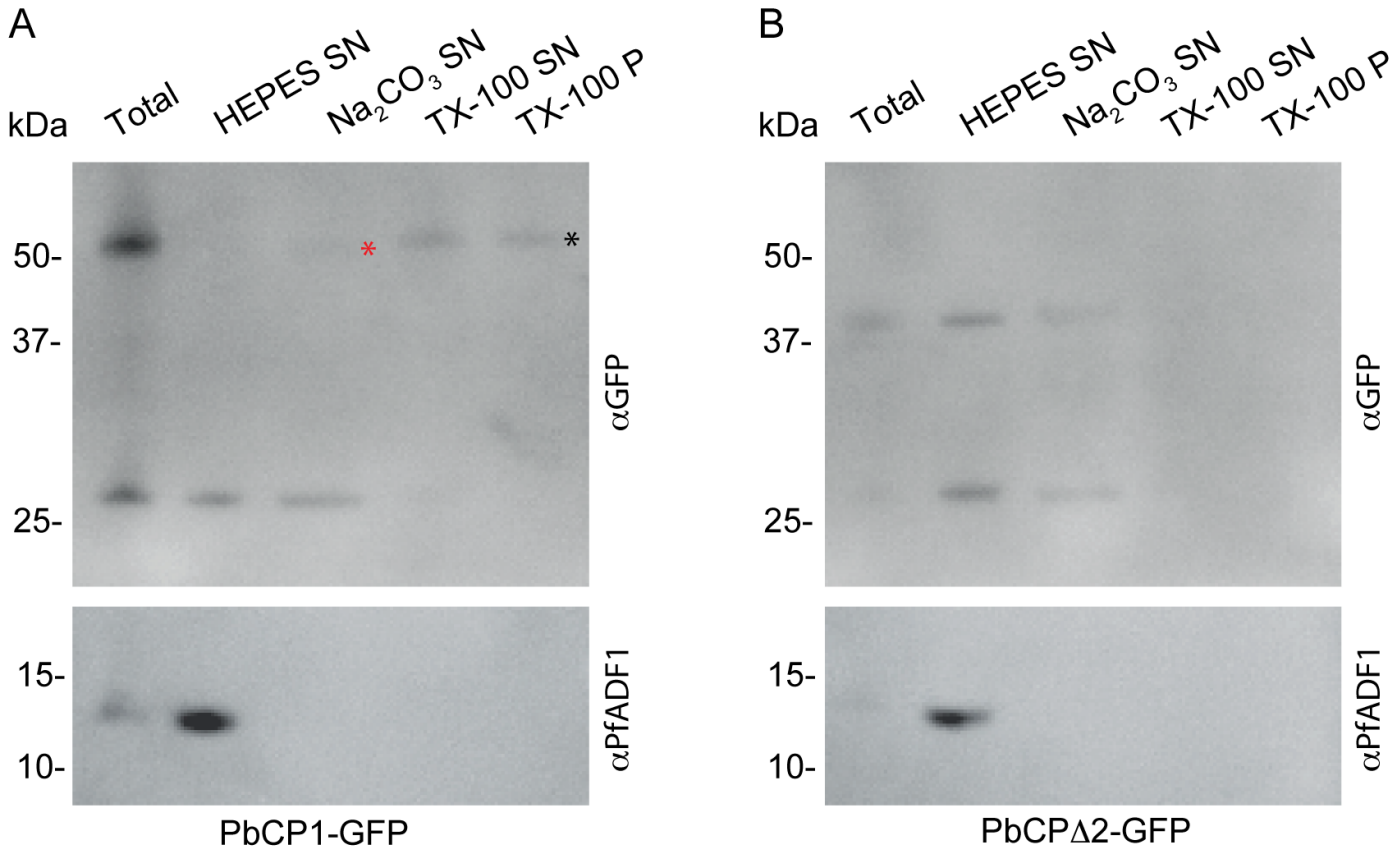
PbCP1-GFP is a membrane-bound protein. (A) PbCP1-GFP mainly associates with the Triton X-100 soluble (TX-100 SN) and insoluble pellet fractions (TX-100 P) as indicated by an ∼52 kDa protein band (black *) in Western blot analysis. A small fraction is also detected in the supernatant (SN) after Na_2_CO_3_ extraction (red *). (B) The PbCP1Δ2-GFP mutant lacking the 2TMD is soluble. Cross-reactive PfADF1 antibodies detected the soluble protein exclusively in the supernatant after hypotonic lysis at the predicted MW of ∼13 kDa. The ∼27 kDa protein band is indicative of cleaved GFP, which no longer associates with the Triton X-100 soluble or insoluble fractions.

The primary localisation data, trafficking analysis and the predicted 2TMD region of PbCP1 suggested this protein associates with the membranous structures identified by TEM. To confirm this, solubility assays were performed on transgenic PbANKA parasites expressing PbCP1-GFP. For this, iRBCs were hypotonically lysed with 1 mM HEPES (pH 7.4) and subsequent extractions of the membrane fractions with 100 mM Na_2_CO_3_ and 1% Triton-X-100 revealed that whilst a small proportion was detected in the Na_2_CO_3_ soluble fraction, the majority of PbCP1-GFP was found in the Triton-X-100 soluble fraction and the final pellet after TX-100 extraction (indicated by asterisks, Figure 9A). These findings confirm the prediction of the 2TMD region in PbCP1-GFP and its tight association with membranous structures. In contrast, PbCP1Δ2-GFP, which did not localise to the extra-parasitic structures, was mainly detected in the supernatant after hypotonic lysis (Figure 9B). As an additional control, both immunoblots were probed with cross-reactive antibodies to the soluble protein *P. falciparum* actin depolymerising factor 1 (PfADF-1) [29], which was exclusively found in the supernatant after hypotonic lysis (Figure 9A–B).

**Figure 10:**
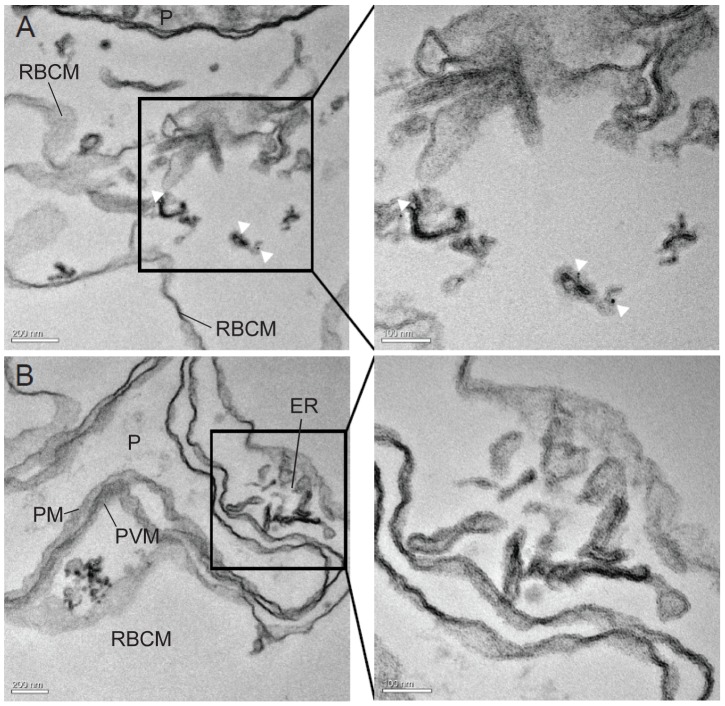
PbCP1-GFP localises to the parasite-induced structures. (A) IEM was performed on the PbCP1-GFP expressing cell line to localise the protein at the ultrastructural level. Anti-GFP conjugated gold particles decorate the extra-parasitic structures (white arrow heads), indicating that PbCP1-GFP associates with these parasite-induced membranous compartments. (B) In contrast, no labeling of the erythrocytic endoplasmatic reticulum (ER) could be detected. Enlargement of the regions boxed in the panels A and B are shown to their right. The parasite (P), parasite plasma membrane (PM), the parasitophorous vacuole membrane (PVM and the red blood cell membrane (RBCM) are indicated.

Finally, the localisation of PbCP1-GFP was also examined at the ultrastructural level by immuno-electron microscopy (IEM) of permeabilised, immunogold-labeled iRBCs. This revealed specific labeling of the parasite-induced structures with anti-GFP coupled gold particles (Figure 10A), indicating that PbCP1 does indeed associate with these structures. In contrast, no labeling of the reticular network was observed (Figure 10B).

## Discussion

Protein export is crucial to the virulence and survival of malaria parasites, thus investigations into the mechanisms behind protein export are of great importance as these pathways provide excellent targets for drug intervention. Several major discoveries over the recent years have accelerated the analysis and our understanding of this vital process in *P. falciparum* (for reviews, see [2], [30]), however, in order to investigate protein export in the context of malaria-associated pathologies, animal models of infection are required. Many studies have been undertaken to analyse pathologies associated with cerebral malaria and sequestration using the murine malaria model (for reviews, see [31], [32]) yet the mechanisms behind these processes are still poorly understood and several differences in the pathology of the human (in particular *P. falciparum*) and rodent infection has raised the question about the applicability of murine malaria models. This, however, highlights the necessity to further decipher the molecular players, interactions and pathways in rodent malaria species to determine their relevance for malaria-associated studies.

In this study, we aimed to gain a deeper insight into the ‘exportome’ of the rodent malaria parasite *P. berghei* [1] and the mechanisms behind protein export. Based on the prediction of a PEXEL motif [1], we identified nine novel *P. berghei* exported proteins that localised to the RBC cytosol (Figures 1, 2, 3, Table S2). This includes the predicted soluble protein Pb114540, in which only a shorter version lacking the last 66aa could be expressed and exported into the host cell. Likewise, another candidate (Pb021580-GFP) could not be expressed under our conditions although analysis of truncated versions of this protein was not examined further. It is interesting to note that despite the prediction of TMDs in the majority of the remaining exported proteins, localisation to the RBC surface was not observed. This may have been due to an adverse effect of the C-terminal GFP reporter masking the putative TMD region of those exported proteins. To address this possibility, we introduced a penta-glycine linker [14] between the C-terminal end of Pb140070, Pb000080, Pb124710 and the GFP to extend the region between the putative TMD and the reporter, however, the resulting GFP chimera were either not expressed (suggesting possible toxicity) or extension did neither improve the level of export nor alter its localisation (data not shown). It should also be mentioned that whilst PbCP1-GFP (with only 11aa between the putative 2TMD region and the reporter) could localise to the discrete structures in the RBC cytosol, Pb070060-GFP, Pb021540-GFP and Pb031630-GFP with ≥20aa between the predicted TMDs and the GFP did not reveal the same phenotype as observed for PbCP1 and IBIS1 nor did they export to the RBC surface. Thus, to confirm the export ultimate destination of the investigated chimeric proteins, specific antibodies against the endogenous protein and/or fusion proteins with smaller epitope tags will have to be generated. Nonetheless, these experiments have validated that all of the expressed proteins, which contain both typical and atypical PEXEL motifs can at least be exported into the iRBC.

Three of the exported proteins, PbCP1-GFP, Pb136550-GFP and Pb114540_F3_-GFP also localised to highly dynamic punctuate structures within the RBC cytosol (Figures 1 and 3, Movie S1) and in agreement with our observations, Pb136550 (IBIS1) has recently been shown to associate with extra-parasitic structures in *P. berghei* iRBCs [18]. Indeed, TEM, electron tomography and IEM revealed parasite-induced vesico-tubular structures of membranous nature in the cytosol of PbANKA iRBCs (Figure 8) to which PbCP1-GFP was found to localise to (Figure 10). The presence of these structures was an intriguing observation and it is tempting to speculate whether they resemble structures similar to the Maurer's clefts or the HSP40-containing J-dots in *P. falciparum*, both of which have also been demonstrated to be highly mobile within the RBC cytosol [8], [33]. Several Maurer's clefts resident proteins have been identified for *P. falciparum*, such as MAHRP1 [34], SBP1 [35], REX1 and REX2 [36], [37] and members of the PfMC-2TM protein family [24] but no obvious orthologues have been described for the rodent malaria parasites. Interestingly, a recent study by Ingmundson and colleagues showed low levels of PfSBP1-GFP export and co-localisation with IBIS1 in *P. berghei* iRBCs [18]. The punctuate localisation pattern of the soluble protein Pb114540_F3_-GFP (as observed in ∼50% of the parasite population) indicates that it might transiently associate with the parasite-induced structures, similarly to the soluble knob-associated histidine-rich protein (KAHRP) *in P. falciparum* [38]. It should be noted that trafficking to the extra-parasitic structures is abolished in the Pb114540_F2_-GFP expressing parasite line, suggesting that the region between aa_184_ and aa_410_ might contain specific information to target the protein to these structures. However, it remains to be examined, whether IBIS1, PbCP1 and Pb114540 indeed co-localise to the same compartments.

Although we were unable to establish a function for PbCP1 given that three independent attempts to genetically disrupt the *pbcp1* gene via double-homologous recombination [39] had failed, these experiments suggest that PbCP1 plays an important role in the survival of the rodent malaria parasite. One hypothesis is that PbCP1 might fulfil similar functions to the SBP1, MAHRP1 and REX1 proteins of *P. falciparum*, which have been shown to be involved in Maurer's clefts architecture and/or trafficking of virulence proteins to the RBC surface [40]–[44]. Similarly to IBIS1 [18], PbCP1-GFP was also found to freely diffuse in the RBC cytosol (Figures 3 and 9), suggesting that it might indeed be associated with other proteins before it reaches its final destination at the vesico-tubular structures. The observation that some of the PbCP1-GFP mutants (e.g. PbCP1Δ1-GFP) were initially found to freely diffuse in the RBC cytosol but at a later time point found to be trafficked to the cleft-like structures indicates that endogenous PbCP1 or other exported proteins might interact with these chimeras to ‘chaperone’ them to the extra-parasitic structures. Further experiments, such as immuno-precipitations, are underway to investigate the interaction of PbCP1 with other proteins to further unravel the function of this protein. Finally, we also addressed the role of the PbCP1 2TMD region and demonstrated that both TMDs of PbCP1 are required to mediate trafficking to the cleft-like structures (Figures 5 and S3).

Western blot analysis revealed a doublet protein band for PbCP1-GFP and PbCP1-3xHA/Strep, respectively, suggesting two forms of PbCP1, of which one may be modified, thus running at a slightly different molecular weight (Figures S2 and S3). Interestingly, both single TMD mutants of PbCP1 run as single protein bands in Western blot analysis, however, PbCP1ΔTMD1-GFP was detected at a higher molecular weight than predicted (Figure S3). It should be noted that a doublet protein band could also be observed for the mutant Pb400_PbCP1TMD_GFP chimera, compared to the single protein band detected for wild-type Pb400-GFP (Figure S5), indicating that the new 2TMD region in the Pb400 chimera may have been modified. These findings suggest a potentially important determinant within the 2TMD region of PbCP1.

A subset of the predicted PEXEL proteins, including PbCP1, contained an unusual tyrosine residue in the last position of the export motif (RxLxY), which has not been validated to direct protein export so far. Examination of the PbCP1 PEXEL residues revealed the tyrosine residue to be dispensable, however, mutagenesis of the R and L residue abolished export and led to the accumulation of the respective GFP chimera at the parasite periphery, such as the parasite plasma membrane and/or the PV(M) (Figure 4). The finding that the arginine and leucine residues are required for efficient export, with the fifth amino acid being dispensable, is consistent with the observations made in *P. falciparum* [9], [10], [19], [21]. Thus, the precise function of the fifth PEXEL residue remains elusive. Nevertheless, a recent study demonstrated that whilst the region downstream of the PEXEL was sufficient to mediate export, the fifth PEXEL residue could indeed rescue export in a scrambled background [19]. Cleavage of the PEXEL motif by plasmepsin V (at the conserved leucine residue) and binding to PI3P have been suggested to play important roles in the initiation of protein export in *P. falciparum* [20], [21], [45], however, the process of PEXEL recognition and cleavage by the rodent plasmepsin V homologue remains to be elucidated.

Extra-parasitic membranous structures in the cytosol of PbANKA iRBCs have also been identified in two recent studies by Curra and co-workers [46] and Ingmundson and colleagues [18]. A *P. berghei* small exported protein (SEP) was found to associate with vesicular structures in the RBC cytosol [46] and TEM of PbANKA iRBCs revealed the presence of tubular structures in the RBC cytosol to which the IBIS1 protein (Pb136550) was shown to localise [18]. These findings, together with our data, emphasize that host cell remodeling is not exclusively restricted to the human pathogens and that the newly identified structures in the RBC cytosol of PbANKA iRBCs might fulfill a similar function in the sorting and trafficking of virulence proteins. Whilst we are just at the beginning of understanding these processes in the rodent malaria parasite, our study provides another insight into the mechanisms behind them and further evidence that host cell remodeling also occurs in the rodent malaria species. In addition, we have identified a subset of novel exported proteins in *P. berghei*, which might be useful to the greater research community to further investigate and evaluate the rodent malaria parasite as an *in vivo* model for *P. falciparum* infections.

## Materials and Methods

### Gene identification and primary sequence analysis

Primary sequence data was obtained from the PlasmoDB website (http://plasmodb.org) using the query parameter ‘Exported Protein with a minimum ExportPred score of 4’ [1]. The resulting candidates were further analysed using different prediction tools on the ExPASy Server (http://expasy.org/tools/) to confirm the presence of signal peptides and/or transmembrane domains (TMD) and were categorised according to the variability of the PEXEL motif. Out of each category, a number of candidates were selected for further analysis (Table S2).

### Plasmid constructs

The full length genes of all selected candidates and the shorter versions of *pb114540* were amplified from *P. berghei* ANKA genomic DNA using the primers as listed in Table S3, thereby inserting StuI/AvrII restriction sites. The penta-glycine and proline-lysine-3x glycine linkers for constructs Pb140070, Pb000080, Pb124710 and Pb400 were introduced upstream of the StuI/AvrII restriction sites within the respective reverse-oligonucleotides (Table S3). The resulting products were cloned into the corresponding sites of vector *ef*1α-PfKAHRP-GFP thereby releasing the *pfkahrp* insert. In order to generate *ef*1-PfKAHRP-GFP, the vector pl0035 [47] was modified by introducing 589 bp of the *ef*1α promoter sequence via KpnI/StuI upstream of the reporter gene (690 bp of *pfkahrp*). *Pfkahrp* was cloned via StuI/AvrII in frame with the *gfp* coding sequence. The *gfp* gene was inserted via AvrII/XhoI, followed by 392 bp of the *pbcam* terminator sequence cloned via XhoI/EcoRV. Episomal expression of the gene of interest is therefore driven under the constitutive *ef*1α promoter. To generate the 3xHA/Strep-tagged version of Pb400, the coding sequence for the GFP tag in the respective GFP construct was removed via AvrII/XhoI and replaced with a combined triple haemagglutinin (HA) and single Strep-II epitope tag. These epitope tags were PCR amplified from pTEX150-HA/Str 3′ [12] using the primers as listed in Table S3, thereby inserting the corresponding restriction sites. In addition, wild type *pbcp1* and *pb400* were subcloned into the TOPO vector pCRII (Invitrogen) for further applications. PbCP1 PEXEL mutants were generated by introduction of the appropriate amino acid substitutions using the QuikChange® Mutagenesis Kit (Stratagene), PbCP1^TOPO^ as template and the primers as listed in Table S3. In order to overexpress wild-type, truncated or modified PbCP1 and Pb400 as C-terminal GFP fusion proteins, the respective inserts were PCR amplified from either PbCP1^TOPO^ or Pb400^TOPO^ using the primer combinations listed in Table S3 and were cloned into the vector *ef*1α-PfKAHRP-GFP via StuI/AvrII. All inserts were sequenced to exclude undesired mutations.

Gene disruption of *pbcp1* was attempted by using the pB3 vector [39] to promote a double-homologous recombination event into the *pbcp1* locus. For this, 1090 bp of the *pbcp1* 5′ end and 1041 bp of the *pbcp1* 3′ end were amplified using primers Pb1 5′-F and Pb1 5′-R and Pb1 3′-F and Pb1 3′-R, respectively (Table S3) and cloned into the appropriate restriction sites of pB3 to flank either side of the *tgdhfr* selection cassette.

### Transfection and selection of transgenic parasites

All experimental procedures, which involved the use of rodents have been approved by the animal ethics committee of Deakin University (approval AWC A97/10). The reference clone15cy1 from the *P. berghei* ANKA strain was used to generate all transgenic parasite lines. Transfection of parasites and selection of the transgenic parasites was performed as previously described [48]. Briefly, nycodenz purified *P. berghei* schizonts were prepared for transfection and DNA constructs were introduced using the Nucleofector® electroporation device (Amaxa). The resulting DNA mixture was injected intravenously into 6–8 weeks old Balb/c mice and drug selection of genetically transformed parasites occurred day 1 post transfection with pyrimethamine (0.07 mg/ml).

### Live cell and immunofluorescence microscopy

Live cell imaging was usually performed between days 7–9 and days 12–14 post transfection. For this, a blood sample was taken, DAPI (4′,6-Diamidine-2′-phenyl-indole-dihydrochloride) added at 0.5 µg/ml and the sample incubated at 37°C for 10 min. GFP-expressing parasites were then imaged with an Olympus IX70 microscope using a 100×/1.37 objective. A CCD camera (XM10) and Cell X software (Olympus) were used to capture images.

For immunofluorescence microscopy, erythrocytes infected with transgenic *P. berghei* parasites were fixed with ice-cold acetone or acetone:methanol (9∶1) for 5 min prior to incubation with either IgG purified rabbit anti-GFP antibodies (1∶2000) or rat anti-HA antibodies (1∶200, Roche) for 1 h, followed by three washes with mouse tonicity PBS and incubation with Alexa Fluor 488 conjugated secondary antibody (1∶2000, Molecular Probes) and DAPI (0.5 µg/ml) for 1 h. Samples were imaged as described above.

### TEM, electron tomography and IEM

Mice were infected with PbANKA parasites and blood was collected at 5–7% parasitemia via cardiac puncture. Infected RBCs containing predominantly trophozoite parasites were collected on a CS column using the VarioMacs system (Miltenyi) and were fixed with 2.5% glutaraldehyde in 0.1 M sodium cacodyle, rinsed and post fixed in reduced osmium tetroxide. The block was stained with aqueous uranyl acetate and Walton's lead aspartate and embedded in LRWhite. Sections for standard EM (80 nm) and tomography (200 nm) were cut and observed in a Tecnai F30 (FEI, Eindhoven, The Netherlands) at 300kV. Tomograms were acquired, reconstructed and segmented as described previously [49].

Immuno-labeling was performed as described previously [50], albeit with small modifications. Harvested iRBCs were rinsed twice in PBS prior to fixation with 2.5% paraformaldehyde in PBS for 10 min. Fixed cells were washed with PBS prior to permeabilisation with EqtII (4HU), rinsed and re-fixed in PBS for further 5 min. The cells were then blocked with 2.5% BSA/PBS, incubated with rabbit anti-GFP antibodies (1∶50, WEHI antibody facility) in 1% BSA/PBS for 2 h and washed in 1% BSA/PBS prior to incubation with protein G conjugated gold particles (6 nm) according to manufacturer's instruction for 1 h (Aurion, The Netherlands). Labeled cells were rinsed twice in 1% BSA/PBS, three times in PBS and subsequently fixed in 2.5% glutaraldehyde in 0.1 M sodium cacodylate. The sample was then embedded in epoxy resin, sectioned to 70 nm and EM was performed as described above.

### Solubility assay

Infected RBCs were collected as described above and underwent hypotonic lysis in 1 mM HEPES (pH 7.4) for 30 min on ice. A sample of the lysate was taken as total protein control and the remainder was subjected to ultracentrifugation at 100 000 g for 30 min at 2°C. The supernatant was collected as hypotonic soluble sample and the pellet was resuspended in 100 mM Na_2_CO_3_ (pH 11.2) to extract peripheral membrane proteins. Incubation and ultracentrifugation was performed as above and the carbonate-soluble fraction collected. The carbonate-insoluble pellet was finally treated with 1% Triton-X-100 to extract integral membrane proteins, incubated at 37°C for 30 min and was needle-passed to facilitate complete resuspension prior to a final ultracentrifugation at 100 000 g for 30 min at 2°C. The supernatant was collected as the detergent soluble fraction and the pellet was resuspended in 1% Triton-X-100. All fractions were supplemented with reducing sample buffer prior to boiling for 5 min at 95°C and Western blot analysis.

### Western analysis

Parasites were harvested from infected rodent blood by saponin lysis with 0.02% saponin for 15 min on ice, followed by centrifugation at 20 000 g for 5 min. After three washes with ice-cold mouse tonicity PBS, complete protease inhibitor (Roche) was added and the pellet resuspended in 1× reducing sample buffer. For immunoblots, *P. berghei* parasite lysates were separated on a 10% SDS-PAGE gel and transferred onto PVDF membrane (Millipore) using SDS transfer buffer with methanol and a wet transfer blotting device (Bio-Rad). Immunoreactions were performed using antibodies in 3% BSA/PBS and detection was performed using ECL (Pierce). Primary antibodies used were monoclonal anti-GFP (1∶2000, Roche), polyclonal anti-PfADF1 (1∶500, a kind gift from J. Baum) and monoclonal anti-HA (1∶1000, Roche). Secondary antibodies were horseradish peroxidase-conjugated goat anti-mouse and goat anti-rabbit antibodies (1∶4000, Jackson IR).

## Supporting Information

Figure S1 Verification of protein export. (A) Immunofluorescence analysis confirms export of Pb070060-GFP and Pb124710-GFP. RBCs infected with transgenic *P. berghei* parasites were fixed with acetone and incubated with anti-GFP antibodies. Immunofluorescence microscopy revealed a uniform GFP signal (green) throughout the infected RBC for both Pb070060-GFP and Pb124710-GFP expressing parasite lines. Parasite nuclei are stained with DAPI (blue) and merged images include bright field. (B) Immunofluorescence analysis of acetone:methanol (90∶10) fixed RBCs infected with transgenic PbANKA parasites expressing the PbCP1-3xHA/Step fusion protein confirms a punctuate expression pattern in addition to a diffuse signal in the RBC cytosol (anti-HA, green). Parasite nuclei are stained with DAPI (blue) and merged images include bright field. Anti-HA antibodies recognise a doublet protein band at the predicted MW of ∼30 kDa for PbCP1-3xHA/Step but not for PbANKA wild-type parasites.
(TIF)

Figure S2 Wild-type PbCP1-GFP and the PEXEL mutants reveal a doublet protein band. Immuno-blots were probed with anti-GFP antibodies. The ∼27 kDa protein bands are indicative of cleaved GFP.
(TIF)

Figure S3 Both predicted TMDs are required to traffic PbCP1 to the extra-parasitic structures. (A) Deletion of either TMD abolished trafficking of the resulting GFP chimera PbCP1ΔTMD1-GFP and PbCP1ΔTMD2-GFP to the discrete structures in live PbANKA parasites but export into the RBC was unaffected. However, ∼5% of the parasites expressing PbCP1ΔTMD1-GFP did not export the GFP chimera into the RBC cytosol. (B) Western blot analysis of truncated PbCP1-GFP expressing parasites. Proteins bands of the predicted MW are detected for PbCP1Δ1-GFP to PbCP1Δ4-GFP (∼44, 38, 32 and 29 kDa, respectively) and PbCP1ΔTMD2-GFP (∼47 kDa) with exception of PbCP1ΔTMD1-GFP running at a slightly higher MW (>50 kDa) than expected (∼48 kDa). A smaller protein fragment (as seen in PbCP1Δ3-GFP) and the ∼27 kDa GFP band are indicative of degradation products.
(TIF)

Figure S4 Verification of Pb400 localisation. (A) Introduction of a linker between the C-terminus of Pb400 and the reporter does not promote trafficking of Pb400 to the extra-parasitic structures. Live microscopy reveals export of the Pb400_PK3xG_-GFP chimera into the host cell cytosol only. GFP fluorescence is indicated by GFP (green) and parasite nuclei are stained with DAPI (blue). Immunoblot analysis confirms expression of the GFP fusion protein at the predicted MW of ∼52 kDa. (B) RBCs infected with transgenic PbANKA parasites expressing Pb400-3xHA/Strep were fixed with acetone:methanol (90∶10) and incubated with anti-HA antibodies. Immunofluorescence microscopy reveals weak punctuate signals within the RBC cytosol in addition to prominent staining within the parasite (green). Parasite nuclei are stained with DAPI (blue) and merged images include bright field. Western blot analysis confirms expression of the 3xHA/Strep chimera at the expected size of ∼30 kDa. Smaller protein bands are indicative of degradation products.
(TIF)

Figure S5 Western blot analysis. Pb400_PbCP1TMD_GFP reveals are doublet protein band around 52 kDa (indicated by asterisks) compared to the wild-type Pb400-GFP. PbCP1_IBIS1TMD_GFP and Pb400_IBIS1TMD_GFP are expressed at the predicted MW of ∼50 kDa.
(TIF)

Table S1 Summary of the initial data set derived from the PlasmoDB database (PLasmoDB 6.0). Presence of a signal peptide is indicated by (+) and N-terminal hydrophobic stretches by (h). The number of predicted transmembrane domains (TMD) and the molecular weight (MW in kDa) of all proteins are given. Putatively exported proteins examined in this study are highlighted in bold.
(DOCX)

Table S2 Summary of investigated proteins and their localisations. Presence of a signal peptide is indicated by (+) and N-terminal hydrophobic stretches by (h). Predicted transmembrane domains (TMD) are indicated by their amino acid (aa) position.
(DOCX)

Table S3 Summary of oligonucleotides used in this study. Restriction sites are shown in lower case and underlined, start ATGs and stop TAAs are shown in bold and mutated bases are in lower case and bold. Introduced linkers are shown in italics.
(DOCX)

Movie S1 The discrete punctuate structures in the RBC cytosol are highly dynamic. Live microscopy was performed on PbCP1-GFP expressing parasites and images taken every 2 sec.
(MOV)

Movie S2 The parasite-induced structures are independent entities. Electron tomograms of serial sections of *P. berghei* iRBCs were collected and show that the discrete membranous structures are not connected to the reticular network.
(MOV)
