# Supplementary figures and images for: The Exported Protein PbCP1 Localises to Cleft-Like Structures in the Rodent Malaria Parasite Plasmodium berghei
Source: PLoS One. 2013 Apr 26;8(4):e61482. doi: 10.1371/journal.pone.0061482 (PMC3637216; doi:10.1371/journal.pone.0061482)

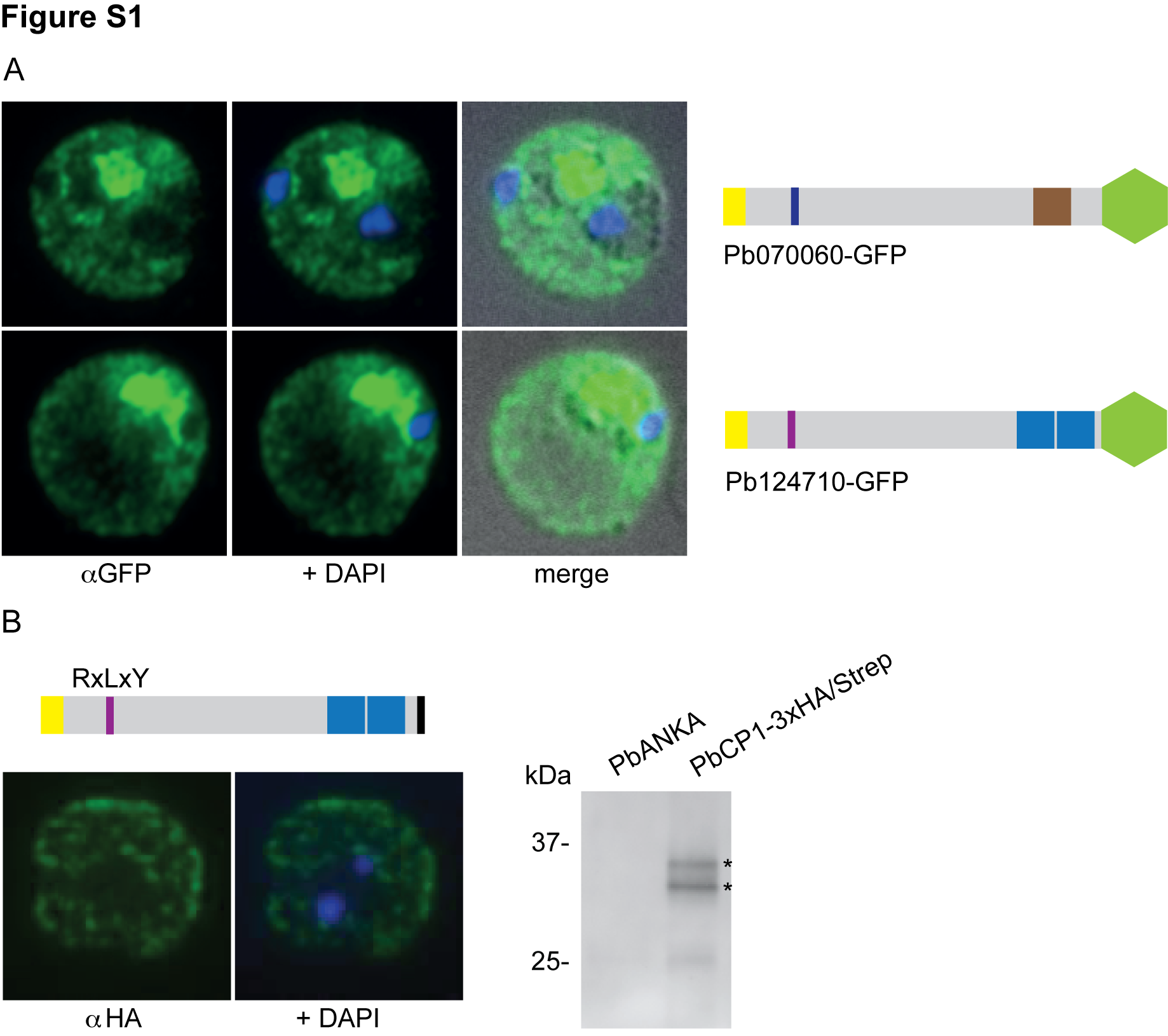

Supplement: Figure S1 — Verification of protein export. (A) Immunofluorescence analysis confirms export of Pb070060-GFP and Pb124710-GFP. RBCs infected with transgenic P. berghei parasites were fixed with acetone and incubated with anti-GFP antibodies. Immunofluorescence microscopy revealed a uniform GFP signal (green) throughout the infected RBC for both Pb070060-GFP and Pb124710-GFP expressing parasite lines. Parasite nuclei are stained with DAPI (blue) and merged images include bright field. (B) Immunofluorescence analysis of acetone:methanol (90∶10) fixed RBCs infected with transgenic PbANKA parasites expressing the PbCP1-3xHA/Step fusion protein confirms a punctuate expression pattern in addition to a diffuse signal in the RBC cytosol (anti-HA, green). Parasite nuclei are stained with DAPI (blue) and merged images include bright field. Anti-HA antibodies recognise a doublet protein band at the predicted MW of ∼30 kDa for PbCP1-3xHA/Step but not for PbANKA wild-type parasites. (TIF) [file pone.0061482.s001.tif]

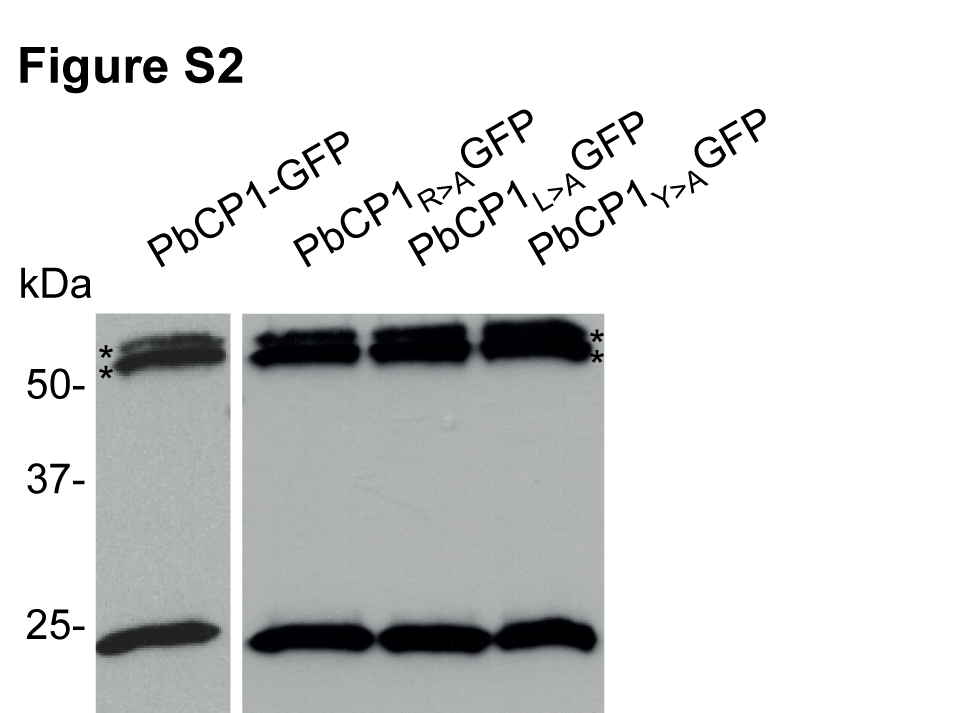

Supplement: Figure S2 — Wild-type PbCP1-GFP and the PEXEL mutants reveal a doublet protein band. Immuno-blots were probed with anti-GFP antibodies. The ∼27 kDa protein bands are indicative of cleaved GFP. (TIF) [file pone.0061482.s002.tif]

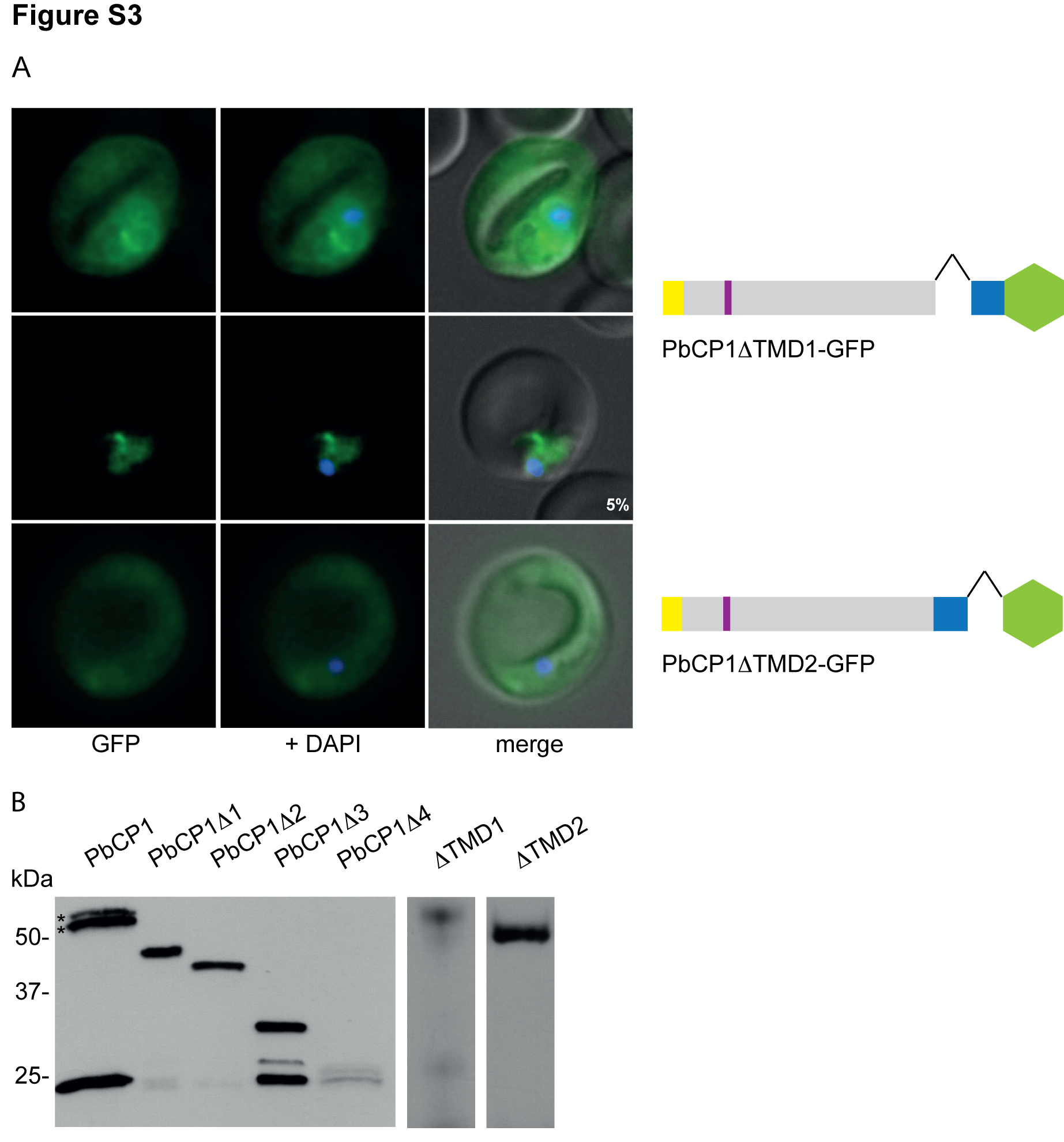

Supplement: Figure S3 — Both predicted TMDs are required to traffic PbCP1 to the extra-parasitic structures. (A) Deletion of either TMD abolished trafficking of the resulting GFP chimera PbCP1ΔTMD1-GFP and PbCP1ΔTMD2-GFP to the discrete structures in live PbANKA parasites but export into the RBC was unaffected. However, ∼5% of the parasites expressing PbCP1ΔTMD1-GFP did not export the GFP chimera into the RBC cytosol. (B) Western blot analysis of truncated PbCP1-GFP expressing parasites. Proteins bands of the predicted MW are detected for PbCP1Δ1-GFP to PbCP1Δ4-GFP (∼44, 38, 32 and 29 kDa, respectively) and PbCP1ΔTMD2-GFP (∼47 kDa) with exception of PbCP1ΔTMD1-GFP running at a slightly higher MW (>50 kDa) than expected (∼48 kDa). A smaller protein fragment (as seen in PbCP1Δ3-GFP) and the ∼27 kDa GFP band are indicative of degradation products. (TIF) [file pone.0061482.s003.tif]

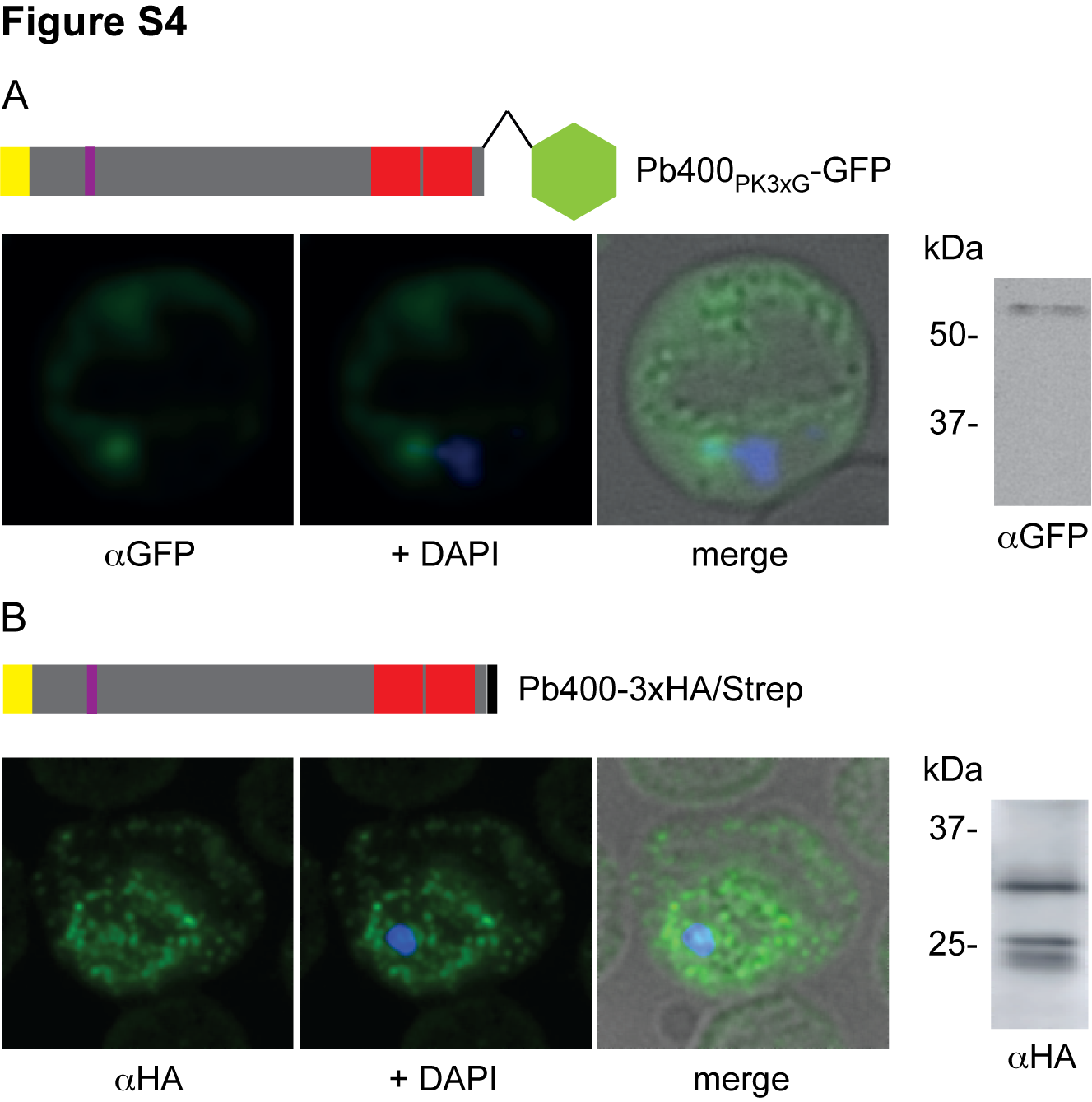

Supplement: Figure S4 — Verification of Pb400 localisation. (A) Introduction of a linker between the C-terminus of Pb400 and the reporter does not promote trafficking of Pb400 to the extra-parasitic structures. Live microscopy reveals export of the Pb400PK3xG-GFP chimera into the host cell cytosol only. GFP fluorescence is indicated by GFP (green) and parasite nuclei are stained with DAPI (blue). Immunoblot analysis confirms expression of the GFP fusion protein at the predicted MW of ∼52 kDa. (B) RBCs infected with transgenic PbANKA parasites expressing Pb400-3xHA/Strep were fixed with acetone:methanol (90∶10) and incubated with anti-HA antibodies. Immunofluorescence microscopy reveals weak punctuate signals within the RBC cytosol in addition to prominent staining within the parasite (green). Parasite nuclei are stained with DAPI (blue) and merged images include bright field. Western blot analysis confirms expression of the 3xHA/Strep chimera at the expected size of ∼30 kDa. Smaller protein bands are indicative of degradation products. (TIF) [file pone.0061482.s004.tif]

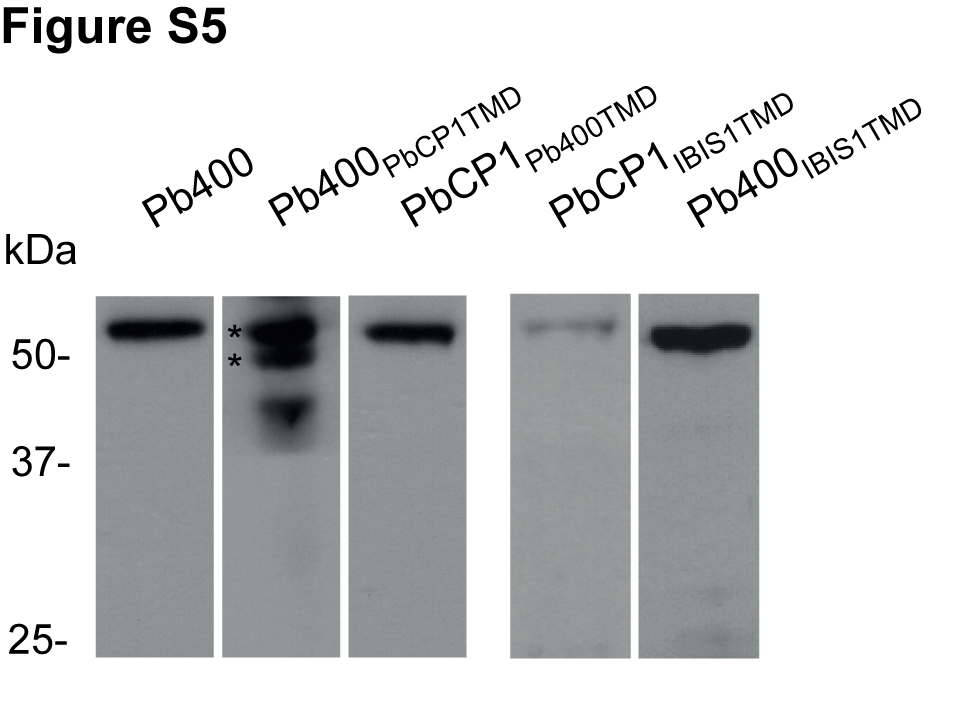

Supplement: Figure S5 — Western blot analysis. Pb400PbCP1TMDGFP reveals are doublet protein band around 52 kDa (indicated by asterisks) compared to the wild-type Pb400-GFP. PbCP1IBIS1TMDGFP and Pb400IBIS1TMDGFP are expressed at the predicted MW of ∼50 kDa. (TIF) [file pone.0061482.s005.tif]
