# Supplementary material for: The Exported Protein PbCP1 Localises to Cleft-Like Structures in the Rodent Malaria Parasite Plasmodium berghei
Source: PLoS One. 2013 Apr 26;8(4):e61482. doi: 10.1371/journal.pone.0061482 (PMC3637216; doi:10.1371/journal.pone.0061482)
Supplement: Table S1 — Summary of the initial data set derived from the PlasmoDB database (PLasmoDB 6.0). Presence of a signal peptide is indicated by (+) and N-terminal hydrophobic stretches by (h). The number of predicted transmembrane domains (TMD) and the molecular weight (MW in kDa) of all proteins are given. Putatively exported proteins examined in this study are highlighted in bold. (DOCX) [file pone.0061482.s006.docx]

**Table S1**

| **Old gene ID**  **(PlasmoDB 6.0)** | **New gene ID**  **(PlasmoDB 8.0)** | **PEXEL motif** | **PEXEL score** | **SP** | **TMD** | **MW (kDa)** | **comments** |
| --- | --- | --- | --- | --- | --- | --- | --- |
|  |  |  |  |  |  |  |  |
| PB108789.00.0 | PbANKA_010020 | RvLsE | 11.93 | + | 2 | 39.5 | Member of *P. yoelii* subtelomeric family PYST-B |
| PB108818.00.0 | PbANKA_100030 | RvLsE | 11.86 | + | 2 | 39.9 |  |
| **PB106275.00.0** | **PbANKA_136550** | **RiLsE** | **11.70** | **h** | **1** | **36.3** | **IBIS-1 (1)** |
| PB402722.00.0 | PbANKA_010020 | RvLsE | 11.51 | + | 2 | 39.5 | Identical to PB108789.00.0 |
| **PB108030.00.0** | **PbANKA_140070** | **RhLaE** | **11.17** | **h** | **2** | **20.0** | **Conserved rodent malaria protein** |
| PB106994.00.0 | PbANKA_070070 | RiLsS | 11.04 | h | - | 24.8 |  |
| PB000439.00.0 | PbANKA_072150 | RnLnE | 10.26 | h | 1 | 261.5 |  |
| **PB103567.00.0** | **PbANKA_021580** | **RiLaD** | **10.08** | **+** | **2** | **29.0** | **PbCP1 paralog**  **Member of *P. yoelii* subtelomeric family PYST-B** |
| PB108101.00.0 | PbANKA_104030 | RiLaD | 9.91 | + | 1 | 28.3 | Member of *P. yoelii* subtelomeric family PYST-B |
| **PB106995.00.0** | **PbANKA_070060** | **RyLsE** | **9.84** | **+** | **1** | **26.1** |  |
| PB402966.00.0 | PbANKA_114540 | RnLsE | 9.28 | + | - | 55.2 | Identical to PB106385.00.0 |
| **PB106385.00.0** | **PbANKA_114540** | **RnLsE** | **9.08** | **+** | **-** | **55.2** | **Homologous to surface protein in *Borrelia burgdorferi***  **Identical to PB402966.00.0 according to new annotation** |
| PB103457.00.0 | PbANKA_124590 | RlLaE | 8.93 | + | 1 | 51.0 | Phage fibre protein superfamily |
| PB000319.03.0 | PbANKA_120060 | RiLaS | 8.88 | h | - | 251.0 |  |
| **PB101512.00.0** | **PbANKA_021540** | **RiLaD** | **8.79** | **+** | **1** | **23.0** | **Member of *P. yoelii* subtelomeric family PYST-B** |
| PB102673.00.0 | PbANKA_021560 | RiLaD | 8.42 | + | 2 | 30.2 | Member of *P. yoelii* subtelomeric family PYST-B |
| **PB108495.00.0** | **PbANKA_000080** | **RtLaD** | **8.29** | **+** | **2** | **28.6** | **PbCP1 paralog**  **Member of *P. yoelii* subtelomeric family PYST-B** |
| PB000565.03.0 | PbANKA_010930 | KiLyE | 8.09 | h | 6 | 33.3 | SNARE associated Golgi protein |
| PB103476.00.0 | PbANKA_124620 | RiLvD | 8.04 | n.a. | n.a. | n.a. | Partial annotation  Identical to PB107841.00.0 according to PlasmoDB 8.0 |
| PB107841.00.0 | PbANKA_124620 | RiLvD | 8.04 | n.a. | n.a. | n.a. | Partial annotation  Identical to PB103476.00.0 according to PlasmoDB 8.0 |
| PB106913.00.0 | PbANKA_010060 | RfLvE | 7.77 | + | 1 | 18.9 | Conserved rodent malaria protein |

**Table S1**

| **Old gene ID**  **(PlasmoDB 6.0)** | **New gene ID**  **(PlasmoDB 8.0)** | **PEXEL motif** | **PEXEL score** | **SP** | **TMD** | **MW (kDa)** | **comments** |
| --- | --- | --- | --- | --- | --- | --- | --- |
|  |  |  |  |  |  |  |  |
| PB000247.02.0 | PbANKA_143230 | KsLaS | 7.46 | + | - | 20.8 | CelTOS (cell traversal protein for ookinetes and sporozoites) |
| **PB104040.00.0** | **PbANKA_031630** | **RiLaY** | **7.36** | **+** | **1** | **32.2** |  |
| PB402997.00.0 | PbANKA_114660 | RtLaD | 6.97 | + | 2 | 28.3 | Member of *P. yoelii* subtelomeric family PYST-B  T-SNARE protein family |
| PB101458.00.0 | PbANKA_146500 | RsLsE | 6.59 | + | - | 97.5 | Putative PYST-C2 homologue |
| PB108856.00.0 | n.a. | RiLsE | 6.24 | n.a. | n.a. | n.a. | Partial annotation |
| PB100936.00.0 | PbANKA_030060 | RnLaE | 5.82 | h | 1 | 72.5 |  |
| PB108171.00.0 | PbANKA_030440 | RiLgD | 5.78 | + | 1 | 21.6 | MSP4/5 |
| PB402817.00.0 | PbANKA_124600 | RiLaY | 5.73 | + | 1 | 26.7 | Member of *P. yoelii* subtelomeric family PYST-B |
| **PB403061.00.0** | **PbANKA_124660** | **RiLaY** | **4.80** | **+** | **2** | **29.0** | **PbCP1**  **Member of *P. yoelii* subtelomeric family PYST-B** |
| PB403104.00.0 | n.a. | RiLaY | 4.79 | n.a. | n.a. | n.a. | Partial annotation |
| PB107834.00.0 | PbANKA_000400 | RiLaY | 4.76 | + | 2 | 28.3 | Highest homology to PbCP1 (Pb400)  Member of *P. yoelii* subtelomeric family PYST-B |
| PB102226.00.0 | PbANKA_140040 | RiLeE | 4.10 | + | 1 | 30.5 | Member of *P. yoelii* subtelomeric family PYST-B |
| PB108348.00.0 | PbANKA_122900 | RnLsE | 4.08 | h | - | 126.3 | Glycoprotein (MG2) |
| PB102443.00.0 | PbANKA_072280 | RiLaY | 4.08 | + | 2 | 28.9 | Member of *P. yoelii* subtelomeric family PYST-B |
| PB103795.00.0 | PbANKA_072260 | RiLeE | 4.03 | + | 1 | 29.9 | Member of *P. yoelii* subtelomeric family PYST-B |
| **PB403086.00.0** | **PbANKA_124710** | **RiLsY** | **4.01** | **+** | **2** | **28.4** | **Member of *P. yoelii* subtelomeric family PYST-B** |

1. Ingmundson A, Nahar C, Brinkmann V, Lehmann MJ, Matuschewski K. The exported *Plasmodium berghei* protein IBIS1 delineates membranous structures in infected red blood cells. *Mol* *Microbiol.* 2012 Mar;83(6):1229-43.
