# Supplementary material for: The Exported Protein PbCP1 Localises to Cleft-Like Structures in the Rodent Malaria Parasite Plasmodium berghei
Source: PLoS One. 2013 Apr 26;8(4):e61482. doi: 10.1371/journal.pone.0061482 (PMC3637216; doi:10.1371/journal.pone.0061482)
Supplement: Table S2 — Summary of investigated proteins and their localisations. Presence of a signal peptide is indicated by (+) and N-terminal hydrophobic stretches by (h). Predicted transmembrane domains (TMD) are indicated by their amino acid (aa) position. (DOCX) [file pone.0061482.s007.docx]

**Table S2**

| **gene ID** | **SP** | **PEXEL** | **TMD (aa)** | **localisation** |
| --- | --- | --- | --- | --- |
|  |  |  |  |  |
| PbANKA_114540 | + | RnLsE | - | RBC cytosol & punctuate expression in RBC cytosol |
| PbANKA_070060 | + | RyLsE | 1 (172-194) | faint RBC cytosol |
| PbANKA_136550 | h | RiLsE | 1 (271-292) | RBC cytosol & punctuate expression in RBC cytosol |
| PBANKA_140070 | h | RhLaE | 2 (125-147, 149-171) | RBC cytosol |
|  |  |  |  |  |
| PbANKA_021540 | + | RiLaD | 1 (151-173) | RBC cytosol |
| PBANKA_000080 | + | RtLaD | 2 (196-218, 224-246) | RBC cytosol |
| PbANKA_021580 | + | RiLaD | 2 (195-212, 217-236) | not expressed |
|  |  |  |  |  |
| PbANKA_124660 | + | RiLaY | 2 (198-215, 220-239) | RBC cytosol & punctuate expression in RBC cytosol |
| PbANKA_124710 | + | RiLsY | 2 (191-213, 215-237) | prominent ER/PV(M), faint RBC cytosol |
| PBANKA_031630 | + | RiLaY | 1 (222-244) | prominent ER/PV(M), faint RBC cytosol |
