# Supplementary material for: The Exported Protein PbCP1 Localises to Cleft-Like Structures in the Rodent Malaria Parasite Plasmodium berghei
Source: PLoS One. 2013 Apr 26;8(4):e61482. doi: 10.1371/journal.pone.0061482 (PMC3637216; doi:10.1371/journal.pone.0061482)
Supplement: Table S3 — Summary of oligonucleotides used in this study. Restriction sites are shown in lower case and underlined, start ATGs and stop TAAs are shown in bold and mutated bases are in lower case and bold. Introduced linkers are shown in italics. (DOCX) [file pone.0061482.s008.docx]

**Table S3**

| **Oligo name** | **Sequence (5’-3’)** | **construct** |
| --- | --- | --- |
|  |  |  |
| EF5'-F | CGGggtaccAGCTTAATTCTTTTCGAGCTCTTTATG | ef1α-PfKAHRP-GFP |
| EF5'-R | CGGctcgagaggcctGATCCCCCTATGTTTTATAAAATT |  |
| CAM3'-F | CGGctcgagATTATTAATATATATGAATATATATACATCG |  |
| CAM3'-R | CGGgatatcGACCATATAAGAATTAACCCTTTAC |  |
| GFP5'-F | CCACCATCACCATcctaggAGTAAAGGAGAAGAACTTTTCACTG |  |
| GFP3'-R | CGGctcgagTTATTTGTATAGTTCATCCATGCCAT |  |
| KAHRP-F | CGGaggcct**ATG**AAAAGTTTTAAGAACAAAAATACTTTG |  |
| KAHRP-R | TTACTcctaggATGGTGATGGTGGTGATGGTGTTC |  |
| PB403061-F | GCGaggcct**ATG**AGAGTCAGTATTTTAAAATATGTTC | Pb124660-GFP (PbCP1) |
| PB403061-R | GCGcctaggTTTTGGTATTTTTTTTAATTTAAAGTTAC |  |
| PB403086-F | GCGaggcct**ATG**AGAGTCAGTATTTTAAAATATGTTC | Pb124710-GFP |
| PB403086-R | GCGcctaggTATTTTTCTTAATTTAATGGAATATTTAATAAG |  |
| PB106385-F | GCGaggcct**ATG**AATACCTATTCAAAATTTTCAAAAATG | Pb114540-GFP |
| PB106385-R | GCGcctaggGCTTTGTATGTCCTTCAAAAACTGCTTAATG |  |
| PB106385F_2_-R | GCGcctaggGGTACCTTGGTCGCCACTTC | Pb114540_F2_-GFP |
| PB106385F_3_-R | GCGcctaggTTGGCGCATCTTGTTATGAGC | Pb114540_F3_-GFP |
| PB101512-F | GCGaggcct**ATG**AGAATCAGTATTTTAAAATATGTTC | Pb021540-GFP |
| PB101512-R | GCGcctaggTTTTAATTTAAAGCGATTATTTCTGG |  |
| PB103567-F | GCGaggcct**ATG**AGAGTCAGTATTTTAAAATGTGTTC | Pb021580-GFP |
| PB103567-R | GCGcctaggTTTTGATATTTTTTCTAATTTACGTTCAC |  |
| PB106995-F | GCGaggcct**ATG**TGTAGTTTAGTTAACATTGTTTCTTG | Pb070060-GFP |
| PB106995-R | GCGcctaggTTTATAAAGTGGAATATTGTTCCTATTGGG |  |
| PB108495-F | GCGaggcct**ATG**AGAGTCAGTATTTTAAAATGCGTTC | Pb000080-GFP |
| PB108495-R | GCGcctaggTTTTGATATGCTTTCTAATTTAAAGATAC |  |
| PB104040-F | GCGaggcct**ATG**AAAAAATCAAATATTTTTAATAAAATATTATTGC | Pb031630-GFP |
| PB104040-R | GCGcctaggGCGGGAATATTTTTTCATTATTTTTCTTATTTTATG |  |
| PB106275-F | GCGaggcct**ATG**GCTCGTAATTGCGAATGCAAAAAGATAG | Pb136550-GFP |
| PB106275-R | GCGcctaggCATAGGTTTTGCTCTACAAAATATGTTAG |  |
| PB108030-F | GCGaggcct**ATG**AATACTGATAAATTTTCGCAAATTTCG | Pb140070-GFP |
| PB108030-R | GCGcctaggCGAAGAATTTATATGAATATAATACAATAC |  |
| Pb1d1-R | GCGcctaggCAGGATGTATTTGATTGCTTCTTTTCT | PbCP1Δ1-GFP |
| Pb1d2-R | GCGcctaggTTTTTTTATTATTTTTTTATTCTCTATCGGCTGTATTGCTAATTCATC | PbCP1Δ2-GFP |
| Pb1d3-R | GCGcctaggTGTTTTACTTCCTTTATGCTTCTTTATATG | PbCP1Δ3-GFP |

**Table S3**

| **Oligo name** | **Sequence (5’-3’)** | **construct** |
| --- | --- | --- |
|  |  |  |
| Pb1d4-R | GCGcctaggATATGCTAATATCCTATTATTTCTAAAGTT | PbCP1Δ4-GFP |
| Pb1TM-S | ACATCAAGTAATCGTCATATGAAATCAAAATTGACTAAA | Pb400_PbCP1TMD_-GFP |
| Pb1TM-AS | GATTGCTTCTTTTCTATATTTTTTAGTCAATTTTGATTTCAT |  |
| PB107834-R | GCGcctaggTATTTTTTTTAATTTAAAGGAACATTTAATA | Pb400-GFP |
| Pb107TM-S | TGCATCAAGTAATTGCTATATGAAATCAAAATTGACTAAA | PbCP1_Pb400TMD_-GFP |
| Pb107TM-AS | CTGATTAATTCTTTTTTAATTTTTTTAGTCAATTTTGATTTCAT |  |
| Pb1_R>A_-S | CTTTAGAAATAAT**gcc**ATATTAGCATATGCAGATAACC | PbCP1_R>A_-GFP |
| Pb1_R>A_-AS | GGTTATCTGCATATGCTAATAT**ggc**ATTATTTCTAAAG |  |
| Pb1_L>A_-S | CTTTAGAAATAATAGGATA**gcc**GCATATGCAGATAACC | PbCP1_L>A_-GFP |
| Pb1_L>A_-AS | GGTTATCTGCATATGC**ggc**TATCCTATTATTTCTAAAG |  |
| Pb1_Y>A_-S | CTTTAGAAATAATAGGATATTAGCA**gcc**GCAGATAACC | PbCP1_Y>A_-GFP |
| Pb1_Y>A_-AS | GGTTATCTGC**ggc**TGCTAATATCCTATTATTTCTAAAG |  |
| Pb15'-F | GCGatcgatGTATATATGATATATGCCAAGAAG | PbCP1KO (5’ flank) |
| Pb15'-R | CGGaagcttGAGATCATGGTGATAACTTGA |  |
| Pb13'-F | CGCggatccGCTTTATTATACATGCTTAAATGGCT | PbCP1KO (3’ flank) |
| Pb13'-R | GCGtctagaGATATCGTTGTGTCATCGAACTTCG |  |
| Pb1dTMD2-R | GCGcctaggGCTCGGCAACCCTATTATTGGTATCG | PbCP1ΔTMD2-GFP |
| Pb1dTMD1-S | GAAATTACATCAAGTAATCGTCATGGGTTGCCGAGCTTAATGATACTAC | PbCP1ΔTMD1-GFP |
| Pb1dTMD1-AS | GTAGTATCATTAAGCTCGGCAACCCATGACGATTACTTGATGTAATTTC |  |
| Pb107-AS | CTTTTTAAATATTTTATCTCATTTTGCATATAGCAATTACTTGATGCAA |  |
| PB106275TM-S | GAAATTACATCAAGTAATCGTCATATGTTAACAAAAGTCTTTGTTGATAATTTG | PbCP1_IBIS TMD_-GFP |
| PB106275-AS | CAAATTATCAACAAAGACTTTTGTTAACATATGACGATTACTTGATGTAATTTC |  |
| PB106275TM-S2 | TTGCATCAAGTAATTGCTATATGTTAACAAAAGTCTTTGTTGATAATTTG | Pb400 _IBIS TMD_-GFP |
| PB106275-AS2 | CAAATTATCAACAAAGACTTTTGTTAACATATAGCAATTACTTGATGCAA |  |
| PB107834-R2 | cctagg*TCCTCCTCCTTTGGG*TATTTTTTTTAATTTAAAGGAACATTTAAT | Pb400_PK3xG_-GFP |
| PB403086-R2 | cctagg*CCCTCCTCCTCCTCC*TATTTTTCTTAATTTAATGGAATATTTAATAAG | Pb124710_5xG_-GFP |
| PB108495-R2 | cctagg*CCCTCCTCCTCCTCC*TTTTGATATGCTTTCTAATTTAAAGATAC | Pb000080_5xG_-GFP |
| PB108030-R2 | cctagg*CCCTCCTCCTCCTCC*CGAAGAATTTATATGAATATAATACAATAC | Pb140070_5xG_-GFP |
| HA-F | cctaggATGGCATACCCGTACGAC | Pb400-3xHA/Strep |
| Strep-R | ggactcgag**TTA**TTTTTCAAATTGTGGATGTGA |  |
